# Multifunctional Metal–Organic Framework/Alkali-Etched Silicon Carbide Composite for Efficient Strontium Adsorption and Ciprofloxacin Removal

**DOI:** 10.1021/acsami.6c08717

**Published:** 2026-07-13

**Authors:** Yongxin Lei, Zhencong Liu, Wenjie Qin, Zihan Li, Dongci Wei, Santosh K. Tiwari, Xinpeng Wang, Yanqiu Zhu, Nannan Wang, Haiyan Mou

**Affiliations:** † State Key Laboratory of Featured Metal Materials and Life-Cycle Safety for Composite Structures, MOE Key Laboratory of New Processing Technology for Nonferrous Metals and Materials, School of Resources, Environment and Materials, 615902 Guangxi University, Nanning 530004, China; ‡ Institute for Disaster Management and Reconstruction, Sichuan University-The Hong Kong Polytechnic University, Chengdu 610065, China; § Centre for New Materials & Surface Engineering, Department of Chemistry, NMAM Institute of Technology, 49605 Nitte University, Nitte, Karnataka 574110, India; ∥ Faculty of Environment, Science and Economy, University of Exeter, Exeter EX4 4QF, U.K.

**Keywords:** Peroxymonosulfate, Silicon carbide, ZIF-67, ZIF-8, Metal−organic frameworks, Adsorption, Strontium

## Abstract

With the acceleration of global industrialization, chemical residues of radionuclides and organic pollutants in water bodies have become an increasingly severe issue. This study developed a ternary heterogeneous structural material, ZIF-8@ZIF-67/AE-SiC, which served as a dual-functional material combining both nuclide adsorption and organic pollutant degradation capabilities. Alkali-etched silicon carbide (AE-SiC) was introduced as a functional carrier, combining the confinement effect of ZIF-8 with the redox activity of ZIF-67 to enhance surface activity and mass-transfer efficiency. On the one hand, ZIF-8@ZIF-67/AE-SiC exhibited a maximum theoretical adsorption capacity of 52.07 mg/g for Sr. The adsorption process was well described by the pseudo-first-order kinetic model and the Freundlich isotherm, and the material exhibited good selectivity under competitive ions. On the other hand, ZIF-8@ZIF-67/AE-SiC removed over 95% of ciprofloxacin (CIP) within 20 min, and showed good efficiency in removing low concentrations (100 μg/L) of CIP. It maintained high-efficiency degradation across a wide pH range (5–11) and temperature range (15–45 °C), and exhibited broad-spectrum degradation capabilities of over 85% for other pollutants. The synergistic effects of radical and nonradical pathways dominated by SO_4_^•–^ and ^1^O_2_ enable rapid decomposition of pollutants. The structural stability was enhanced due to the stabilizing effect of the Si–O-M bonds and the supporting role of the shell–core structure, resulting in a 60–80% reduction in Zn/Co leaching concentrations compared to pure ZIFs. This work provides a new approach for developing efficient and multifunctional water treatment materials.

## Graphical abstract

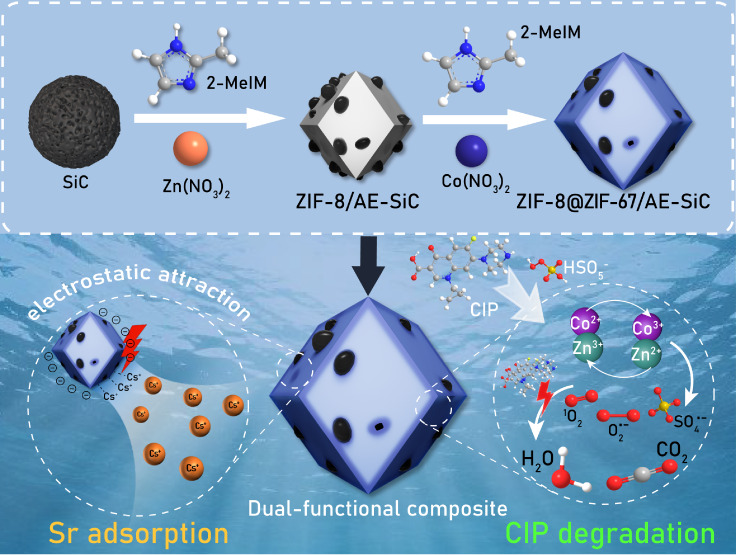

## 1 Introduction

Along with rapid scientific and technological development, increasing amounts of organic pollutants and radioactive contaminants have been released into aquatic environments, posing serious threats to ecosystem stability and human health.^1,2^ Among the available treatment technologies, advanced oxidation processes (AOPs) have gradually gained focus by virtue of their unique reaction mechanism, which centers on the generation of highly reactive radicals (e.g., OH•, SO_4_^•–^, etc.) that convert pollutants into nontoxic or low-toxic substances, thus realizing the elimination of pollution risk from the source.^3,4^ Since SO_4_^•–^ has a similar redox potential to OH•, can be used in a wider pH range (2–9), and has a longer half-life (SO_4_^•–^, 30–40 μs. OH•<1 μs).^5^ Therefore, Sulfate Radical-based Advanced Oxidation Processes (SR-AOPs) have become a hot research topic in environmental catalysis. For SR-AOPs, cobalt ions are recognized as the most efficient catalytic mode. However, the homogeneous Co^2+^/PMS system suffers from secondary contamination by transition metal leaching.^6^ In view of this, researchers have proposed a solid-loading strategy for nonhomogeneous cobalt-based catalysts, which involves activating PMS by immobilizing Co^2+^ in carrier materials (e.g., metal oxides, carbon-based matrices) to form a stable heterogeneous interface using a solid-phase electron-transfer mechanism.^7^

Apart from organic pollutants, radioactive nuclides generated during the nuclear fuel cycle have become another major environmental concern.^8,9^ Radionuclides generated therein (^137^Cs, ^90^Sr, ^235^U, ^106^Ru, etc.) are released into the ecosystem, further contaminating natural environments such as soil, water sources, and air and posing significant risks to human health and environmental safety.^10^ Among them, ^90^Sr, as one of the most hazardous radionuclides, has attracted widespread attention because of its long half-life, solubility, and high radioactivity and toxicity.^11^ At present, among the treatment methods for radionuclides, adsorption is considered to be one of the most promising methods for removing radionuclides. It demonstrates high removal efficiency, ease of operation, and excellent economic benefits.^12^ Traditional adsorbent materials such as zeolites, polymers, and montmorillonites have been used for Sr removal from nuclear waste.^13–15^ According to reports, nanomaterials possess larger specific surface areas and richer adsorption sites relative to conventional materials.^16^ However, most of the catalyst or adsorbent samples studied so far are monofunctional, which limits the development of the potential capability of the samples. Therefore, the development of bifunctional materials with both adsorption and degradation capabilities is an important step in responding to current pollutant treatment requirements.

Metal–organic frameworks (MOFs) consist of metal clusters and organic ligands, which have emerged as promising candidates for environmental remediation because of their high porosity, adjustable pore size, high density of open sites, topological diversity, and tunability.^17,18^ Among them, zeolitic imidazolate frameworks (ZIFs), with highly tunable pore structure, abundant surface functional groups, and metal active sites, show unique advantages in the field of PMS activation and adsorption.^19–21^ However, conventional ZIF-based catalysts still face bottlenecks such as easy passivation of active sites, low mass transfer efficiency, and insufficient cycling stability in practical use. Recent studies have shown that the construction of bimetallic MOFs can optimize the redox cycling kinetics of different valence metal ions through the intermetallic synergistic effects, markedly improving the catalytic system’s reactivity and enhancing PMS activation.^22^ Wang et al. successfully synthesized bimetallic MOF nanosheets called FeCo-BDC, which were superior to the single-metal Co-BDC and Fe-BDC for PMS activation, enabling efficient oxidation of organic substances in water.^23^ Dong et al. constructed Ce/Zr-UiO-66 composites, and the bimetallic ZIFs had high enrichment efficiency for chloramphenicol as well as high sensitivity for its detection.^24^ These findings suggest that constructing bimetallic MOFs is an effective strategy for improving the reactivity and functionality of MOF-based materials. Despite these advances, most reported MOF-based systems have been developed for either adsorption or catalysis individually. Moreover, the limited accessibility of active sites and inefficient mass transport continue to restrict their practical performance. Therefore, developing multifunctional MOF composites with high catalytic activity, strong adsorption capability, and abundant, accessible active sites remains a significant challenge.

Silicon carbide (SiC) is a typical ceramic material characterized by excellent chemical stability, corrosion resistance, and thermal conductivity.^25,26^ It can be used as an ideal catalyst support, which can effectively reduce the risk of the catalytic reaction process.^27,28^ The construction of nanomaterials with specific structures can effectively enhance the specific surface area and the density of active sites on SiC, thus significantly enhancing its degradation performance of SiC. Based on this, we believe that constructing MOFs/SiC interface composites holds promise for simultaneously enhancing both activity and stability. However, dual-performance materials for the efficient removal of radionuclides Sr and organic pollutant CIP are currently understudied.

In this work, a ZIF-8@ZIF-67/AE-SiC ternary heterostructure catalyst was designed and constructed through a multiscale interface engineering strategy. The alkali-etched SiC (AE-SiC) served as a functional carrier, while the ZIF-8@ZIF-67 core–shell structure combined the confinement effect of ZIF-8 with the redox activity of ZIF-67, providing abundant accessible active sites and efficient mass-transfer pathways. The resulting composite was evaluated for the adsorption of Sr^2+^ and PMS-assisted degradation of ciprofloxacin (CIP). The adsorption and catalytic mechanisms were systematically investigated through adsorption experiments, radical-quenching tests, and intermediate product analyses. The environmental adaptability and cycling stability of the samples were further explored. This work provides a new strategy for designing multifunctional MOF-based materials for the synergistic treatment of radioactive nuclides and organic contaminants in complex wastewater systems.

## 2 Experimental Section

### 2.1 Chemicals

The chemicals used in this paper were listed in Text S1

### 2.2 Synthesis Procedures

#### 2.2.1 Preparation of ZIF-8/AE-SiC

First, 6 mmol of Zn­(NO_3_)_2_·6H_2_O with 2.5 mmol of AE-SiC was put into 250 mL of methanol solution to form solution A. 24 mmol of dimethylimidazole was put into 250 mL of methanol solution as solution B. Solution A and B were stirred separately for 30 min, respectively, and then solution B was slowly poured into solution A. The mixture was stirred and allowed to stand for 12 h. The resulting grayish-white precipitate was collected by centrifugation, washed 3 times with methanol, and finally dried under vacuum to obtain ZIF-8/AE-SiC.

#### 2.2.2 Preparation of ZIF-8@ZIF-67/AE-SiC

All the samples prepared above were dispersed in 30 mL of methanol to form a mixed solution, which was termed as Solution A. Six mmol of Co­(NO_3_)_2_·6H_2_O was placed in 60 mL of methanol solution, which was referred to as Solution B. Weigh 24 mmol of dimethylimidazole into 100 mL of methanol solution, which was referred to as Solution C. Subsequently, Solution B was slowly poured into Solution A and stirred for 30 min. Subsequently, Solution C was poured into the above mixed solution. After stirring the mixture at room temperature and allowing it to stand for 24 h, the resulting light purple precipitate was separated by centrifugation, washed 3 times with methanol, and collected as a powder after vacuum drying. This powder was collected as ZIF-67@ZIF-8/AE-SiC, and the average yield was approximately 83.7%. The syntheses of ZIF-8/AE-SiC and ZIF-8@ZIF-67/AE-SiC are shown in Figure 1a. For comparison, the synthesis of ZIF-8, ZIF-67, and ZIF-8@ZIF-67 is listed in Text S2.

**Figure 1:**
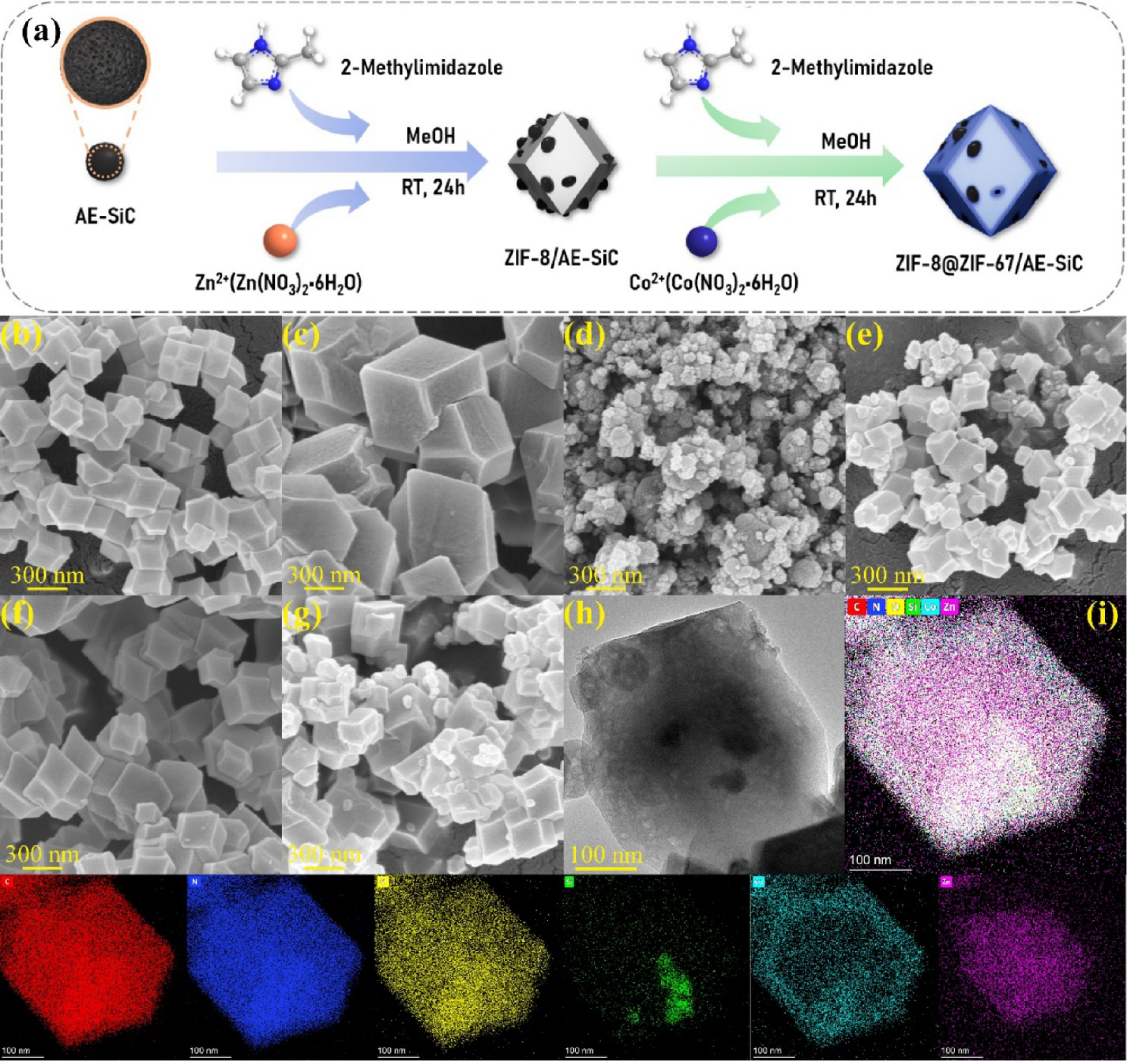
(a) Schematic synthesis of ZIF-8@ZIF-67/AE-SiC materials. SEM images of (b) ZIF-8, (c) ZIF-67, (d) AE-SiC, (e) ZIF-8/AE-SiC, (f) ZIF-8@ZIF-67, and (g) ZIF-8@ZIF-67/AE-SiC. (h) TEM image and (i) TEM-EDS mapping of ZIF-8@ZIF-67/AE-SiC.

### 2.3 Adsorption Experiments

The chemicals used in this paper are listed in Text S3.

### 2.4 Degradation Experiments

The chemicals used in this paper are listed in Text S4.

### 2.5 Characterization

The chemicals used in this paper are listed in Text S5.

## 3 Results and Discussion

### 3.1 Characterization

SEM was applied to investigate the materials’ morphology. As shown in Figure 1b, the synthesized ZIF-8 possessed a typical rhombic dodecahedral structure with uniform size and smooth surface. The synthesized ZIF-67 was larger in size compared to ZIF-8, which is of no small help in synthesizing the shell–core structure of ZIF-8@ZIF-67 (Figure 1c).^29^ The AE-SiC in Figure 1d possessed a smaller particle size, which helped in synthesizing composites with small surface modifications. Some of the AE-SiC was distributed on the surface of the synthesized intermediate ZIF-8/AE-SiC, as can be seen in Figure 1e. Moreover, Figure 1f showed the ZIF-8@ZIF-67 image, which has a slightly larger particle size compared to the pure ZIF-8, and other literature structural features are in high agreement, suggesting the successful composition of the shell-and-core structure. Figure 1g showed the complex ZIF-8@ZIF-67/AE-SiC, with fine particles on the surface partially indicating that AE-SiC was attached to the sample. Furthermore, the morphological analysis of ZIF-8@ZIF-67/AE-SiC was carried out by TEM in Figure 1h. The AE-SiC can be seen embedded on the sample’s surface, which demonstrates the successful complexation of SiC on the sample surface. Figure 1i displayed the results of TEM-EDS mapping, where it can be clearly seen that part of the AE-SiC was embedded on the sample, while the distribution of Zn and Co elements was obvious, yielding the successful synthesis of the shell–core structure.

To investigate the physical phase composition and crystal structure of the prepared samples, the XRD results are shown in Figure 2a. In the magnified images at 2θ = 6–14° (Figure S1), the characteristic diffraction peaks of ZIF-8 in ZIF-8/AE-SiC appeared to be shifted to the right compared with those of pure ZIF-8, and ZIF-8@ZIF-67/AE-SiC appeared to be shifted to the right compared with those of ZIF-8@ZIF-67. This phenomenon of rightward peak shift may be attributed to the interfacial interaction or lattice strain generated when AE-SiC is attached to the surface of ZIFs. As shown by the enlarged plots in Figure S2 with 2θ = 32–40°, ZIF-8@ZIF-67/AE-SiC showed a leftward shift of about 0.33° compared to that in ZIF-8@ZIF-67, which was attributed to the introduction of ZIFs that caused lattice strain in the AE-SiC component as well, which may lead to the formation of chemical bonding structures such as Zn–O–Si or Co–N–C at the interface.^30^ The diffraction peak shift of the AE-SiC substrate was simultaneously detected in the ZIF-8@ZIF-67/AE-SiC system, indicating that the interaction was characterized by bidirectionality, which suggested the formation of a stable composite structure by chemical bonding between the ZIFs and the AE-SiC, rather than a simple physical mixing. These phenomena proved the existence of strong interactions between the components and the successful synthesis of ZIF-8@ZIF-67/AE-SiC.

**Figure 2:**
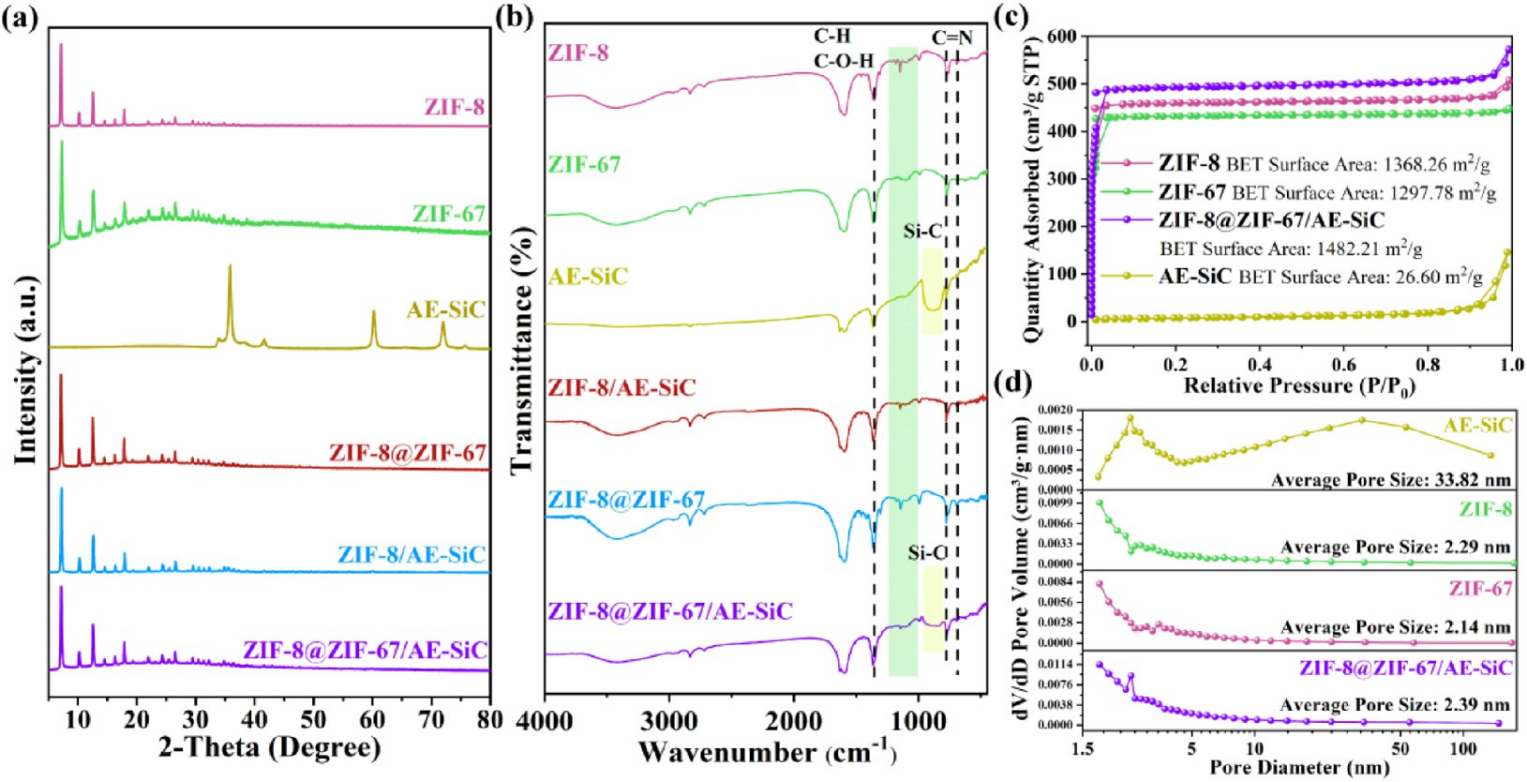
(a) XRD patterns and (b) FT-IR spectra of ZIF-8, ZIF-67, AE-SiC, ZIF-8@ZIF-67, ZIF-8/AE-SiC, and ZIF-8@ZIF-67/AE-SiC. (c) N_2_ adsorption–desorption isotherms and (d) pore size distributions of ZIF-8, ZIF-67, AE-SiC, and ZIF-8@ZIF-67/AE-SiC.

Figure 2b showed the FT-IR spectra of the samples. For ZIF-8@ZIF-67/AE-SiC, the peaks at 1400 cm^–1^ can be seen to be associated with C–H and C–O–H bonds. The characteristic peaks in the interval of 1300–980 cm^–1^ originated from the imidazole ring vibration. The peaks in the interval of 980–800 cm^–1^ were attributed to the Si–C bond vibration, and the peaks at 755 cm^–1^ and 690 cm^–1^ correspond to the C=N bond stretching vibration in 2-MeIm, respectively.^31–33^ The peaks observed at 755 cm^–1^ and 690 cm^–1^ were attributed to the C=N bond stretching vibration and cis-bis-H vibration in 2-MeIm.^34^ The characterized peaks in the ZIF-8@ZIF-67/AE-SiC composites were similar to other materials, which proved their successful synthesis.

As illustrated in Figure 2c, it can be concluded that ZIF-8 and ZIF-67 were typical microporous materials with high specific surface area and high pore volume, which conformed to the structural characteristics of MOFs.^35^ The specific surface area and pore volume of AE-SiC were much lower than those of ZIF materials, and there were almost no micropores, and its pore structure mainly consisted of larger mesopores or macropores. In contrast, ZIF-8@ZIF-67/AE-SiC has the highest BET specific surface area (1482 m^2^/g) and total pore volume (0.885 cm^3^/g), which were slightly higher than those of pure ZIF-8 and ZIF-67. This suggested that the composite process optimized the pore structure of the composites and formed a structure with greater adsorption capacity. In Figure 2d, both ZIF-8 and ZIF-67 exhibited an average pore size of approximately 2 nm, which was a typical microporous material, and the adsorption isotherms also showed typical type I isotherm characteristics. In contrast, AE-SiC exhibited a much larger average pore size of 33.8 nm, which belonged to the mesoporous or macroporous range. Its adsorption isotherm exhibited type IV isotherm characteristics with an obvious hysteresis loop, reflecting the existence of mesoporous structure.^33^ The average pore size of ZIF-8@ZIF-67/AE-SiC (2.39 nm) was slightly larger than that of the pure ZIFs but still mainly in the microporous range. Its t-Plot microporous pore volume occupied a high percentage (0.739/0.885≈83.5%), and the adsorption isotherm rose rapidly in the low-pressure region, showing significant microporous characteristics. Meanwhile, there was a significant rise in adsorption in the high relative pressure region (*P*/*P*_0_ > 0.8) with a very small hysteresis loop, suggesting the existence of some mesoporous or macroporous structures in addition to the main micropores. This suggested that the ZIF material formed a hierarchical pore structure after composite with AE-SiC, and its material partially complied with Murray’s mass transfer theorem. The high specific surface area (1482 m^2^/g) meant that ZIF-8@ZIF-67/AE-SiC could provide more surface atoms or defect sites. These sites were essential for adsorption and activation of PMS to generate highly reactive radicals. Although ZIF-8@ZIF-67/AE-SiC materials were mainly microporous, the hierarchical pore structure with mesoporous or macropores could significantly improve the rates of inward diffusion of the reactants and outward diffusion of the products, which made the internal active sites more accessible and utilizable, and overcame the mass-transfer limitations of purely microporous materials.

The chemical composition and bonding state of the samples were characterized by XPS. The XPS survey in Figure 3a showed that the characteristic signals of C 1s (61.66 at%), N 1s (16.43 at%), O 1s (12.03 at%), Zn 2p (0.24 at%), Co 2p (4.55 at%), Si 2p (3.66 at%), and Si 2s (1.43 at%) were simultaneously detected in ZIF-8@ZIF-67/AE-SiC, which demonstrates the successful synthesis of ZIF-8@ZIF-67/AE-SiC composites. The low Zn content was due to the shell–core structure of ZIF-8 encapsulated by ZIF-67, resulting in poor scanning of Zn within ZIF-8. The percentage of Si 2p and Si 2s in the AE-SiC was 21.64 at% and 22.27 at%, respectively, which was comparable to that of the composite’s Si 2s. The difference in the ratio of atomic percentage of Si 2s to Si 2p could be attributed to the variation of Si–O_*x*_ chemical bonding as well as the effect of Co 3s satellite peaks.

**Figure 3:**
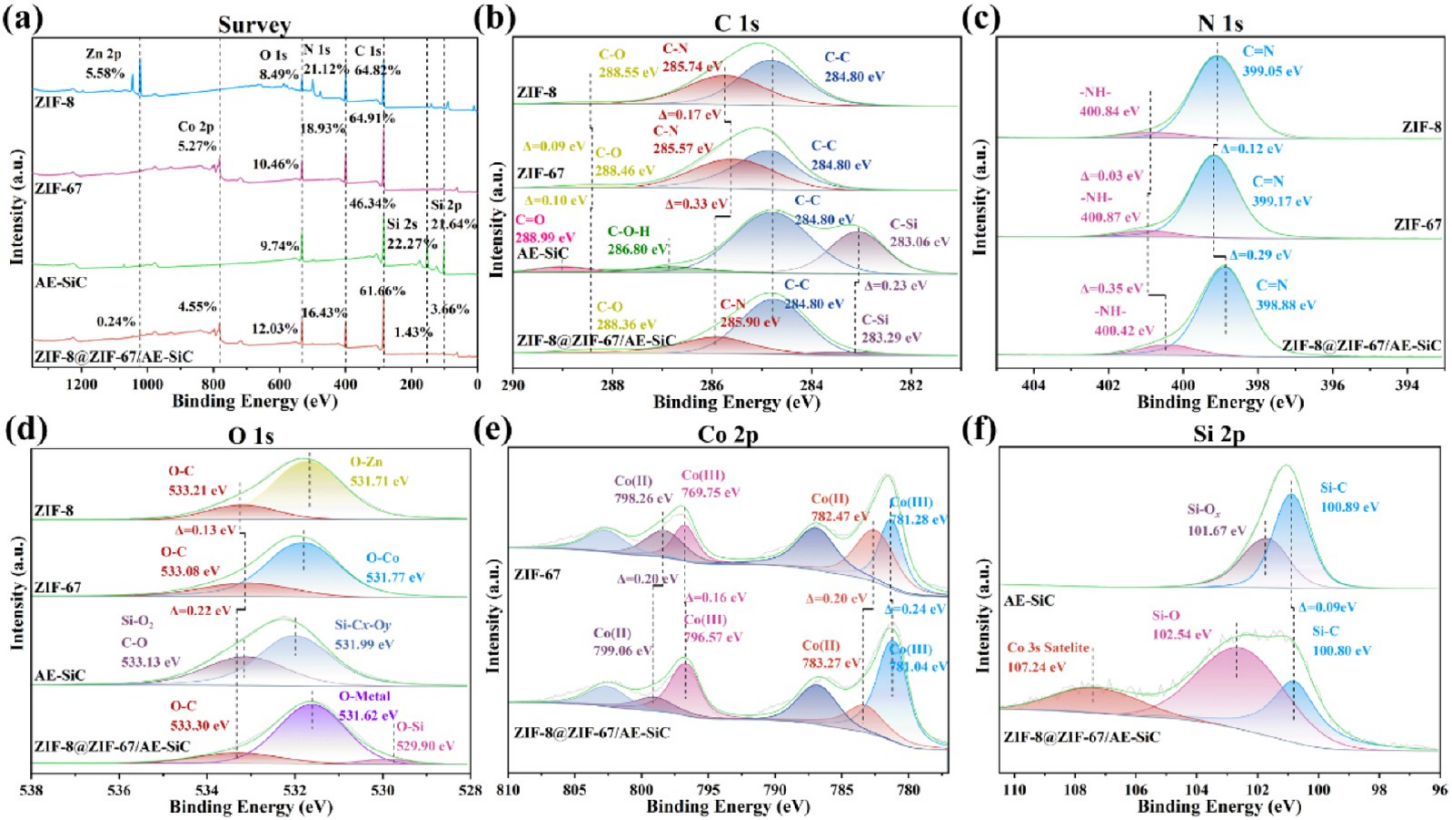
XPS spectra of (a) survey, (b) C 1s, (c) N 1s, (d) O 1s, (e) Co 2p, and (f) Si 2p of as-prepared composites.

In the C 1s spectrum (Figure 3b), the differences between the 284.80 eV (C–C), 285.74 eV (C–N), and 288.55 eV (C–O) characteristic peaks of ZIF-8 and the 284.80 eV (C–C), 285.57 eV (C–N), and 288.46 eV (C–O) peaks of ZIF-67 were less than 0.3 eV, which reflected the high degree of structural similarity of both MOFs’ high structural similarity.^36,37^ The C 1s spectra of AE-SiC showed characteristic peaks at 283.06 eV, corresponding to C–Si, and 288.99 eV, corresponding to C=O, which were in agreement with previously reported SiC surface oxidation behavior.^30^ The C 1s spectra of the composites retained both the C–N peak from ZIF-67 and the C–Si peak from AE-SiC, and the main peaks were shifted overall by 0.2–0.4 eV toward lower binding energies. This indicated that the interfacial electron transfer increased the electron density of the carbon skeleton. As shown in Figure 3c, the N 1s spectra could be deconvoluted into C=N and −NH– peaks of the ZIF-8@ZIF-67/AE-SiC, which exhibited a significant shift compared to other samples. This indicated that the metal–ligand synergism can effectively regulate the electronic structure of the material.^38^ O 1s spectral analysis further revealed the individual material interactions in Figure 3d. The 531.71 eV (O–Zn) peak of ZIF-8 and the 531.77 eV (O–Co) peak of ZIF-67 merged into a 531.62 eV broadening peak (O-Metal) in the ZIF-8@ZIF-67/AE-SiC complexes, which, combined with the emerging O–Si characteristic peak at 529.90 eV, confirmed that the metal nodes of the ZIFs were bridged with the AE-SiC through Si–O–M (M = Zn/Co) bonds. The 101.67 eV (Si–O_*x*_) peak of the AE-SiC in the Si 2p spectra was shifted in the complexes to 102.54 eV (Si–O) with a displacement of 0.87 eV, further supporting the formation of Si–O-Metal bonds. The evolution of the Co 2p spectrum provided evidence of a change in the coordination environment of the metal centers (Figure 3e). The characteristic peaks of Co­(III) (781.28 eV), Co­(II) (782.47 eV), and the satellite peaks (786.92 eV) in ZIF-67 were in agreement with the literature, whereas they were shifted in the complexes to 781.04, 783.27, and 786.80 eV, with the binding energy positively shifted by 0.16–0.24 eV, indicating that the electron cloud density decreased after the coordination of the Co center with the Si–O group.^39^ Meanwhile, the formation of Si–O-M bonds confirmed the existence of chemical bonding between AE-SiC and ZIFs in Figure 3f. The interfacial electron redistribution induced a systematic displacement of the multicomponent binding energy. This evidence indicated the successful synthesis of ZIF-8@ZIF-67/AE-SiC.

### 3.2 Adsorption Analysis

The adsorption performances of different adsorbents were investigated, as shown in Figure 4a. ZIF-8/AE-SiC exhibited a higher adsorption capacity than either ZIF-8 or AE-SiC, which might be because the rapid mass-transfer channels of AE-SiC attracted more Sr^2+^ to the surface of the pollutant. ZIF-8@ZIF-67 exhibited an adsorption capacity of 34.8 mg/g, owing to the core–shell structure providing the adsorbent with more abundant adsorption channels. Moreover, the adsorption capacity of ZIF-8@ZIF-67/AE-SiC was 42.7 mg/g, which meant that the addition of ZIF-67 further enhanced the adsorption performance. Therefore, ZIF-8@ZIF-67/AE-SiC was selected as the representative adsorbent for the subsequent adsorption evaluations.

**Figure 4:**
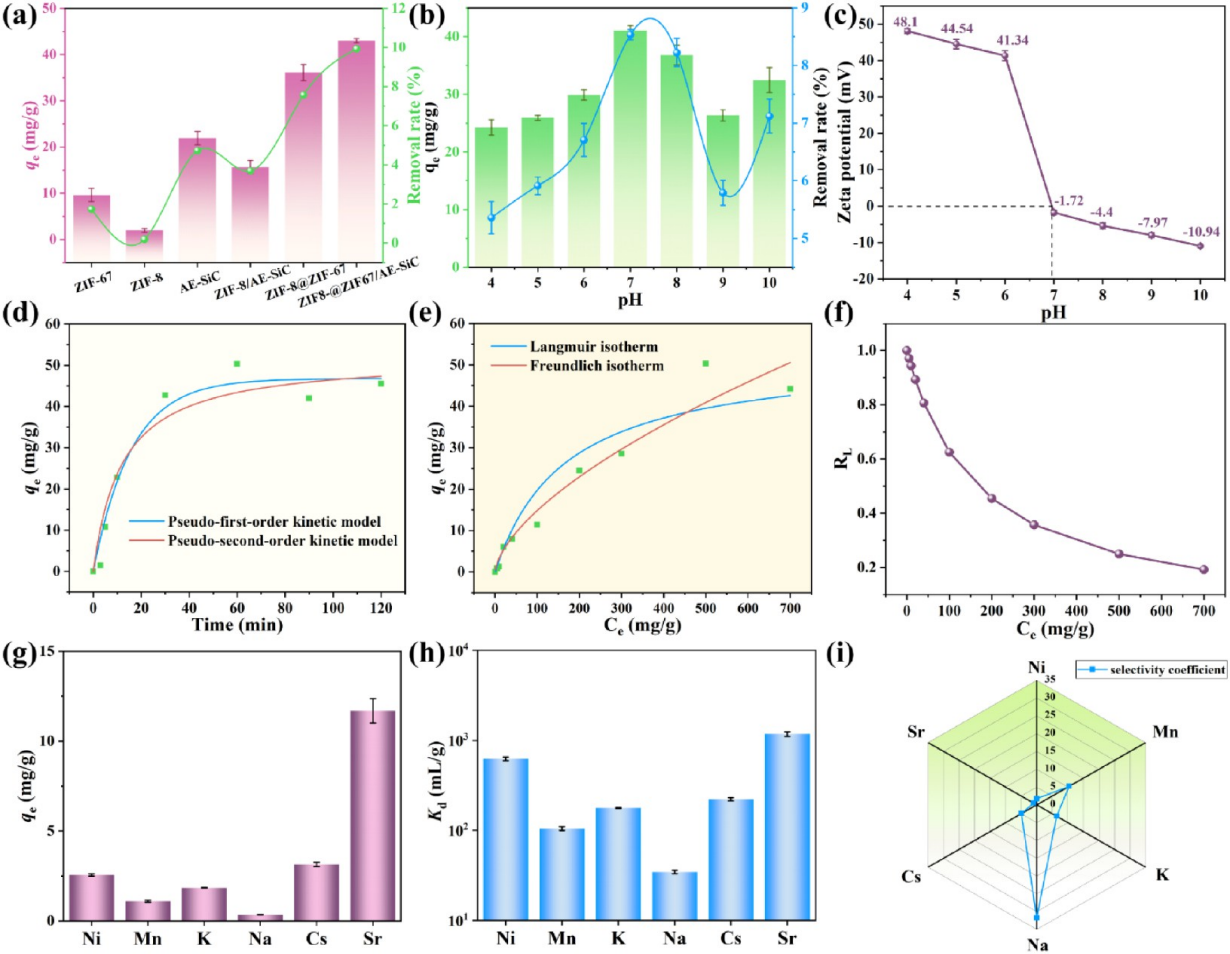
(a) Adsorption effect of different adsorbents. (b) Effect of pH on ZIF-8@ZIF-67/AE-SiC. (c) Zeta potential, (d) adsorption kinetics diagrams, (e) adsorption isotherm diagrams, (f) *R*_L_ of ZIF-8@ZIF-67/AE-SiC. Coexisting ion effect on the adsorption of (g) *q*_e_, (h) *K*_d_, and (i) selectivity coefficient of ZIF-8@ZIF-67/AE-SiC (experimental condition: *m*_absorbent_ = 6 mg, *V* = 3 mL, *C*_0_ = 100 ppm, *t* = 1 h, pH = 7, and RT).

As shown in Figure 4b, the pH value of the adsorption environment exerted a significant impact on adsorption performance. Under acidic conditions, the adsorption capacity of the adsorbent increased with rising pH. When pH = 7, the adsorbent exhibited the highest adsorption capacity and removal efficiency, at 40.5 mg/g and 8.60%, respectively. When pH > 7, the adsorption capacity decreased. To further investigate the effect of pH on adsorption, the zeta potential of the sample was measured at different pH values, as shown in Figure 4c. Under acidic conditions, the sample surface carried a positive charge, and excess H^+^ ions caused electrostatic repulsion, resulting in poor adsorption performance of the adsorbent toward Sr^2+^. When the sample was in the pH range of 7–10, it exhibited a negative charge. The positively charged Sr^2+^ ions might interact electrostatically with the sample, increasing its adsorption capacity. However, higher pH values did not further enhance the adsorption capacity, possibly because under alkaline conditions, excessive −OH groups might bind with free Sr^2+^ ions, reducing the number of available ions in the solution. Therefore, to evaluate the adsorbent’s maximum adsorption capacity and determine optimal adsorption conditions, subsequent adsorption experiments were conducted using an adsorption solution with a pH of 7.

Investigation of adsorption kinetics was crucial for further understanding the rate and potential interaction mechanisms of the adsorbent ZIF-8@ZIF-67/AE-SiC during the uptake of target ions. As shown in Figure 4d and Table 1, the adsorption capacity of ZIF-8@ZIF-67/AE-SiC for Sr^2+^ increased over time, reaching adsorption equilibrium after 30 min due to depletion of the adsorption sites. Additionally, the *R* ^2^ values for the pseudo-first-order and pseudo-second-order kinetic models for Sr^2+^ were 0.973 and 0.946, respectively. Therefore, the pseudo-first-order kinetic model better fitted the Sr^2+^ adsorption process, indicating that physical adsorption played a dominant role.^40^

**Table 1:** Kinetic Data of ZIF-8@ZIF-67/AE-SiC for the Removal of Sr^2+^

Model	Parameters	Sr^2+^
Pseudo-first-order kinetic model	*k*_1_ (/min)	0.0631
	*q*_e_ (mg/g)	42.7269
	*R* ^2^	0.9725
Pseudo-second-order kinetic model	*k*_2_ (/min)	0.0016
	*q*_e_ (mg/g)	52.0679
	*R* ^2^	0.9458

At concentrations ranging from 5 to 300 ppm, the adsorbent’s adsorption capacity exhibited a positive correlation with the concentration of the Sr solution (Figure 4e). At low concentrations, the surface of ZIF-8@ZIF-67/AE-SiC possessed sufficient adsorption sites. As the Sr^2+^ concentration increased, a stronger driving force enabled Sr^2+^to overcome the mass transfer resistance of ZIF-8@ZIF-67/AE-SiC, thereby increasing the collision frequency between Sr^2+^ and the adsorption sites, resulting in an enhanced adsorption capacity of ZIF-8@ZIF-67/AE-SiC. At a concentration of 500 ppm, the adsorption sites on ZIF-8@ZIF-67/AE-SiC tend to reach saturation, exhibiting adsorption capacity approaching equilibrium, with an adsorption capacity of 50.31 mg/g. Based on the fitting results, the *R* ^2^ values for the Langmuir model and the Freundlich model were 0.909 and 0.944, respectively (Table 2). Therefore, the Freundlich model better described the adsorption process of the adsorbent. This indicated that Sr^2+^ adsorption on ZIF-8@ZIF-67/AE-SiC was primarily multilayer adsorption.^41^ Additionally, the simulated results showed that the Freundlich model constant *n* was 1.589 (*n* > 1), indicating that ZIF-8@ZIF-67/AE-SiC exhibited strong adsorption performance toward Sr^2+^. As shown in Figure 4f and Table S1, the *R*_L_ values were all within the range of 0–1 and decreased with increasing initial concentration.^42^ This indicated that the adsorbent ZIF-8@ZIF-67/AE-SiC had a certain adsorption affinity for Sr^2+^.

**Table 2:** Adsorption Isotherm Data of ZIF-8@ZIF-67/AE-SiC for the Removal of Sr^2+^

Model	Parameters	Sr^2+^
Langmuir	*q*_m_	52.7068
	*K*_L_	0.0060
	*R* ^2^	0.9105
Freundlich	*K*_F_	0.8194
	*R* ^2^	0.9444
	*n*	1.5890

It was crucial to investigate the competitive adsorption between coexisting metal cations and target ions in an adsorption solution. The distribution coefficient (*K*_d_) and selectivity coefficient of each metal cation were calculated using the equations. The *K*_d_ value reflected the interaction between the metal cation and the adsorbent, with higher values indicating stronger interaction and greater adsorption capacity of the adsorbent for the corresponding metal ion.^43^ The selectivity coefficient reflected the affinity of the adsorbent for the metal ion, with a smaller value indicating stronger affinity. In Figure 4g–i, ZIF-8@ZIF-67/AE-SiC exhibited the highest *q*_e_ and *K*_d_ values of Sr^2+^, while the selectivity coefficient value of Sr^2+^ was the lowest, indicating that ZIF-8@ZIF-67/AE-SiC has the highest selectivity and the strongest affinity for Sr among various cation systems. This preferential adsorption behavior could be attributed to the suitable ionic radius and hydration characteristics of Sr^2+^ compared with those of competing ions, which facilitated the diffusion of Sr^2+^ into the pore channels of the adsorbent. Furthermore, as a divalent cation, Sr^2+^ possesses a higher charge density than monovalent metal ions, resulting in stronger electrostatic interactions toward negatively charged adsorption sites and facilitating the formation of stable coordination complexes with surface functional groups.^44^ The synergistic effects of these factors contribute to the enhanced selectivity of ZIF-8@ZIF-67/AE-SiC toward Sr^2+^ in the presence of competing ions.

To evaluate the adsorption performance of ZIF-8@ZIF-67/AE-SiC toward Sr^2+^, its adsorption behavior was compared to that of previously reported adsorbents (Table S2). Although some materials exhibited higher adsorption capacities under specific conditions, ZIF-8@ZIF-67/AE-SiC achieved an adsorption capacity of 42.7 mg/g and reached adsorption equilibrium within 30 min, demonstrating a significantly faster adsorption rate than that of most reported adsorbents.

### 3.3 Adsorption Mechanism

To investigate the adsorption mechanism of ZIF-8@ZIF-67/AE-SiC toward Sr, the samples were characterized before and after the reaction. As shown in the SEM image of the adsorbent after adsorbing Sr^2+^ (Figure 5a), the morphology of the adsorbent remained unchanged after adsorbing the target metal cations, indicating that the Sr^2+^ adsorption had little impact on the adsorbent and suggesting that the adsorption process was likely dominated by physical interactions. Additionally, as shown in Figure 5(b,c), the peak positions of XRD and FT-IR for the adsorbent remained consistent after the reaction, suggesting that the adsorbent’s structure did not undergo significant changes. For FT-IR, the weakening of peaks might result from the blocking of Sr adsorbed on its surface. This further confirmed that the adsorption process was predominantly governed by physical adsorption. The XPS survey after the reaction is presented in Figure 5d. In the sample after the reaction, a new characteristic peak of Sr was observed at 134.32 eV. For the characteristic peak of N, slight shifts were observed in the C=N and −NH– peaks after adsorption, and no new chemical state peaks appeared. These results indicated that Sr adsorption by the sample occurred primarily through physical adsorption. In the high-resolution Sr spectrum (Figure 5f), the peaks at 133.57 and 135.33 eV correspond to Sr 3d_5/2_ and Sr 3d_3/2_, further proof of the sample’s successful adsorption of Sr. Moreover, the SEM-EDS of the adsorbed sample is shown in Figure 5g. The presence of Sr on the sample surface confirmed the effective adsorption of Sr by ZIF-8@ZIF-67/AE-SiC.

**Figure 5:**
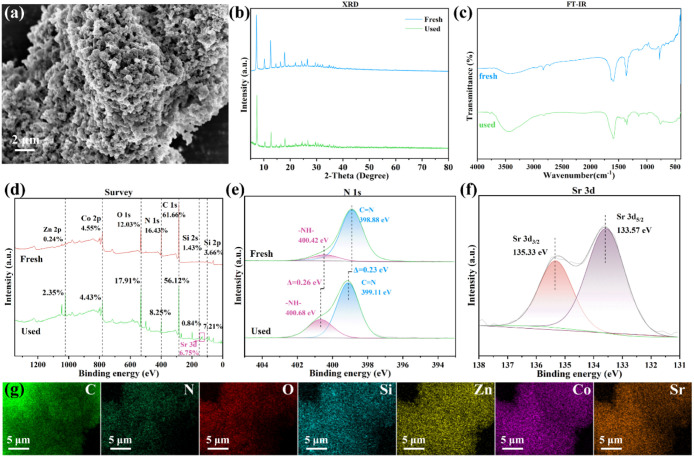
(a) SEM image, (b) XRD pattern, and (c) FT-IR spectra of ZIF-8@ZIF-67/AE-SiC after adsorption. XPS spectra of (d) survey, (e) N 1s, and (f) Sr 3d for ZIF-8@ZIF-67/AE-SiC after adsorption. (g) SEM-EDS of ZIF-8@ZIF-67/AE-SiC after adsorption.

### 3.4 Degradation Analysis

Catalytic degradation experiments were carried out to evaluate the performance of the catalysts, as shown in Figure 6a. The experimental data showed that the blank control group (without catalyst and PMS) and the system containing only PMS had negligible degradation of CIP, with quasi-primary reaction rate constants (*k*_obs_) as low as 0.00083 min^–1^ and 0.00119 min^–1^, respectively, confirming that the self-decomposition effect of CIP or PMS contributed weakly to the removal of pollutants. The same occurred in the AE-SiC+PMS and ZIF-8+PMS systems, where their degradation rates were 10.75% and 11.95%, respectively, which were mainly attributed to the porous properties of AE-SiC and ZIF-8, and their limited removal effects were mainly originated from the physical adsorption of pollutants by the porous structure of the materials. Significantly improved degradation performance of ZIF-8/AE-SiC was observed, which was attributed to the fast mass transfer channel of AE-SiC, which allowed the catalyst to enter the interior of ZIF-8 to achieve the effect of an accelerated adsorption rate. ZIF-67+PMS and ZIF-8@ZIF-67+PMS systems also demonstrated good degradation performance, degrading 78.15% and 80.12% of CIP within 20 min, respectively. However, the ZIF-8@ZIF-67/AE-SiC composite exhibited efficient degradation performance, achieving 71.74% adsorption performance in 20 min in pure adsorption effect without PMS, while its degradation performance reached 95.93% in 20 min with the addition of PMS. This could be attributed to the porous properties of the shell–core structure of ZIF-8@ZIF-67 compounded with AE-SiC, while AE-SiC provided new mass transfer channels, which led to an increase in adsorption efficiency. The ZIF-67 layer on the surface reacted with PMS in a redox reaction, generating a large number of radicals and nonradicals to react with the pollutants. This result reflected the coreaction of adsorption and radical oxidation, which led to the rapid degradation of the pollutants.

**Figure 6:**
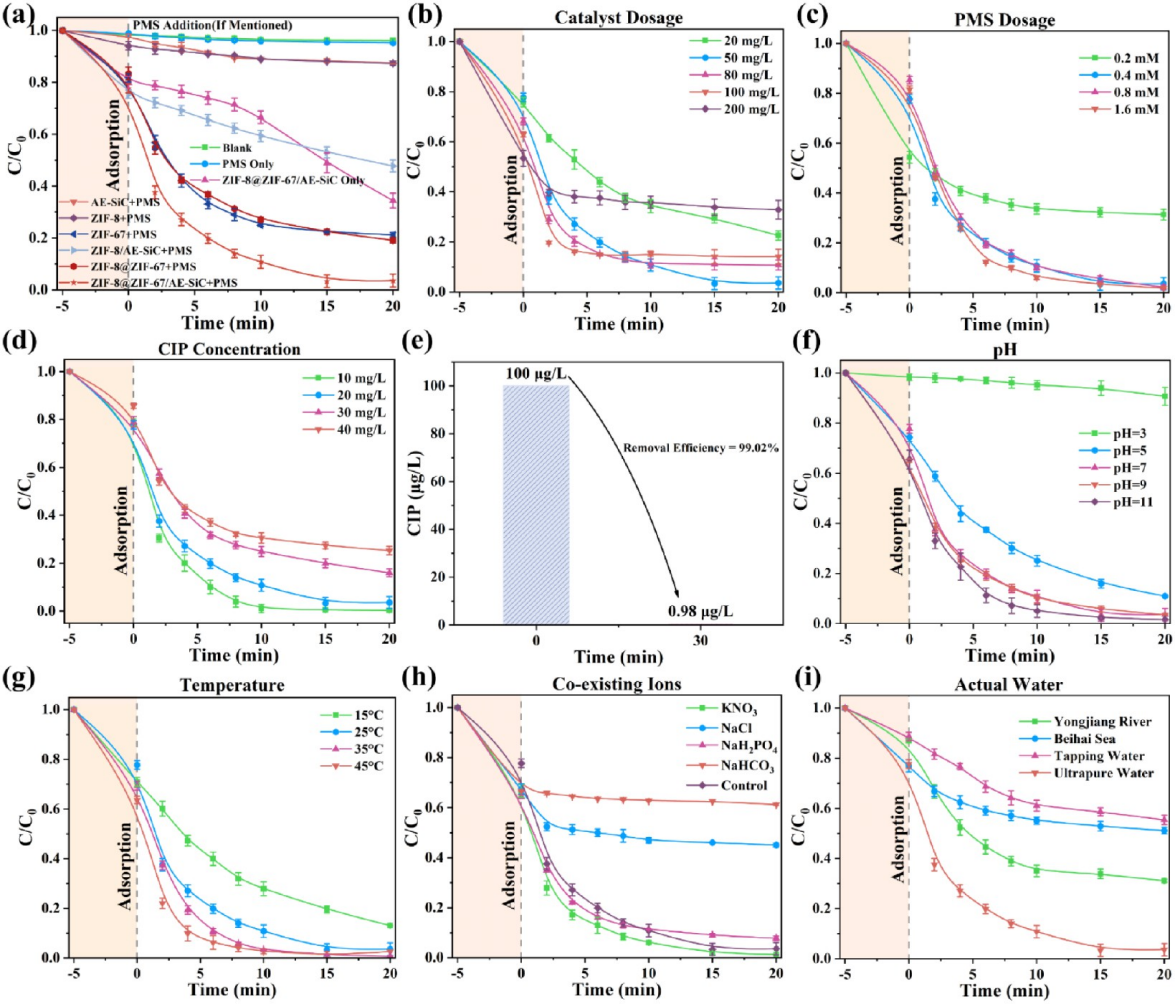
(a) Degradation effect of CIP by using different catalysts. Effect of (b) catalyst dosage, (c) PMS dosage, (d) CIP concentration, (e) low concentration (100 μg/L) of CIP, (f) pH, (g) reaction temperature on CIP degradation, (h) coexisting ions, and (i) different water matrices in the ZIF-8@ZIF-67/AE-SiC+PMS system (experimental conditions: *m*_catalyst_ = 6 mg, PMS= 0.4 mM, *V* = 100 mL, *C*_0_ = 20 mg/L, pH = 7, and RT).

The catalytic behavior of the ZIF-8@ZIF-67/AE-SiC+PMS system was further investigated for CIP degradation under various reaction conditions, with the *k*_obs_ results shown in Figure S3. The results in Figure 6b showed that the catalyst dosage had a significant modulating effect on pollutant removal efficiency. The CIP degradation rate was proportional to the catalyst addition when the catalyst dosage increased from 20 mg/L to 200 mg/L, which was mainly due to the increase in the density of active sites that effectively promoted the activation process of PMS. Meanwhile, in Figure 6c, the PMS dosage was linearly and positively correlated with the *k*_obs_ value in the range of 0.2–1.6 mM, indicating that the increase of PMS concentration favored the continuous generation of radicals and nonradicals. When the initial CIP concentration was increased from 10 mg/L to 40 mg/L, the removal efficiency of the system decreased significantly from 99.45% to 75.94% within 20 min (Figure 6d), which confirmed that a given quantity of PMS and catalysts can only react with a specific quantity of pollutant molecules, and a high concentration of CIP led to a shortage of radicals and nonradicals, thus making the degradation efficiency lower. Given that the environmental concentrations of CIP and other antibiotics were typically on the order of tens to several hundreds of ng/L,^45,46^ the potential for low-concentration degradation by ZIF-8@ZIF-67/AE-SiC was assessed at a CIP concentration of 100 μg/L by HPLC-MS. As shown in Figure 6e, the ZIF-8@ZIF-67/AE-SiC+PMS system achieved a degradation efficiency of 99.02% for trace amounts of CIP. This indicated that the ZIF-8@ZIF-67/AE-SiC+PMS system exhibited good feasibility for treating low-concentration contaminants.

The effect of initial solution pH in CIP degradation is shown in Figure 6f. The *k*_obs_ value significantly increased from 0.00496 min^–1^ to 0.17563 min^–1^ in the pH 3.0–11.0 range (Figure S3e), indicating that the catalyst was more suitable for use under neutral and weakly alkaline conditions. The zeta potential of ZIF-8@ZIF-67/AE-SiC is shown in Figure 4c. The zeta potential gradually decreased from 48.10 mV at pH = 4 to −10.94 mV at pH = 10, with an isoelectric point located near pH = 6.97. CIP possessed two dissociation constants (p*K*_a_) of 6.18 and 8.76.^47^ Under acidic conditions, CIP predominantly existed as CIP^+^. Meanwhile, the catalyst surface became highly protonated, causing electrostatic repulsion between the catalyst and CIP^+^. It further inhibited PMS activation and interfacial electron-transfer processes, thereby reducing the degradation efficiency.^48,49^ As the solution pH increased to alkaline conditions, CIP predominantly existed as CIP^–^. Although the catalyst surface also became negatively charged, the alkaline environment would facilitate PMS activation and promoted the generation of O_2_^•–^ and ^1^O_2_, thereby maintaining a high degradation efficiency.^50–52^

The results of the temperature effect experiments are shown in Figure 6g, where the system exhibited typical thermal activation characteristics. When the reaction temperature was increased from 15 to 45 °C, the *k*_obs_ value grew from 0.08460 min^–1^ to 0.29097 min^–1^ (Figure S3f), and the temperature increase not only enhanced the mass transfer efficiency by promoting molecular thermal motion but also, more importantly, lowered the activation energy barrier for PMS decomposition, which accelerated the radical generation reaction.^53^

To assess the broad-spectrum degradation performance of the catalytic system, tetracycline hydrochloride (TCH), rhodamine B (RhB), and carbamazepine (CBZ) were further selected as representative pollutants for validation in this study. As shown in Figure S4, the degradation rates of TCH, RhB, and CBZ were 87.86%, 99.93%, and 94.91% within 20 min, respectively, confirming that the catalyst has excellent degradation ability for different structural organic compounds containing heterocyclic, polycyclic aromatic hydrocarbons, and drug molecules. Figure 6h demonstrated the effect of ionic concentrations of 10 mM typical inorganic ions (NO_3_^–^, Cl^–^, H_2_PO_4_^–^, HCO_3_^–^) on the degradation efficacy of the ZIF-8@ZIF-67/AE-SiC+PMS system. It was revealed that the addition of 10 mM HCO_3_^–^ significantly inhibited the degradation reaction, with *k*_obs_ being 0.00852 min^–1^, which could be attributed to the reaction of OH• with HCO_3_^–^ to generate low-activity CO_3_•–.^54^ Meanwhile, the pH buffering effect of the carbonate system inhibited the hydroxylation process on the catalyst surface.^55^ When the concentration of Cl^–^ was increased to 10 mM, its inhibitory effect on CIP degradation decreased the *k*_obs_ to 0.02636 min^–1^ (Figure S3h). The combination of Cl^–^ with free radicals to generate low-activity chlorine radicals (Cl^•^, Cl_2_^•–^) while forming Co–Cl complexes on the catalyst surface, resulting to the passivation of the active site.^56,57^ The introduction of NO_3_^–^ enhanced the degradation rate to 98.74%, which was attributed to the accelerated adsorption of CIP by the catalyst by NO_3_–.^58^ H_2_PO_4_^–^ exhibited a unique dual mechanism of action. In the early stage (0–10 min) H_2_PO_4_^–^ reacts with OH• to generate H_2_PO_4_^•^ with high activity, as shown in eq 1, but the later stage (10–20 min)­may be attributed to the generation of stabilizing Co-PO_4_ complexes that inhibit the reaction process, and the final degradation rate was maintained at 92.85%.

1 $$H_{2}{\mathrm{PO}}_{4}^{-}+\mathrm{OH•}\to H_{2}{\mathrm{PO}}_{4}^{•}{+\mathrm{OH}}^{-}$$

Figure 6i compared the effect of different aqueous matrices on the catalytic performance. The complex environment in real water bodies affected the degradation performance of the system. The water body of Yongjiang River showed the same performance as the ultrapure water group in the adsorption stage, but the degradation rate decreased to 48.42% after PMS activation. This decline was primarily attributed to the occupation of catalyst mesoporous channels by dissolved organic matter (DOM) in the river water through π-π stacking, and the possible competitive reaction of humic acid with radicals.^59^ The tap water system presented a stronger inhibition effect, with a final degradation rate of 32.59%. This phenomenon could be attributed to the use of chlorine during wastewater treatment at the waterworks, as its reactions were similar to those observed with added Cl^–^. The corrosion products of the walls in the water mains (Fe^3+^, Al^3+^, etc.) would alter the electronic structure of the catalysts through metal leaching. The extreme inhibitory effect of the North Sea seawater system, on the other hand, stems from the inactivation of the catalytic system triggered by high salinity of Cl^–^, which resulted in a degradation rate of 27.15%. The spatial site-blocking effect caused by colloidal particles in the seawater would lead to a decrease in the degradation and adsorption performance.

Figure S5a demonstrates the cycling stability of the ZIF-8@ZIF-67/AE-SiC. 4-cycle CIP degradation experiments using the recycled catalyst revealed that the degradation rate of about 80% was maintained within 20 min, although the performance in the fourth cycle declined compared with the first. The loss in catalytic activity was attributed to reaction intermediates and contaminant molecules coating the catalyst surface, causing structural changes in the active sites.^39^ Figure S5b,c compares the XRD spectra and FT-IR spectra of fresh and used ZIF-8@ZIF-67/AE-SiC. It was realized that the XRD and FT-IR analyses showed that the intensities and positions of the characteristic peaks of the composites before and after the reaction were basically the same, which confirmed that the crystal structure of the material remained stable during the catalytic process. Figure S5d shows that the catalyst still maintained the complete dodecahedral morphology in the SEM image after the reaction. The EDS surface distribution map in Figure S5e confirmed the homogeneous distribution of the elements, which verified that the material has excellent structural stability and recyclability. The dynamic leaching behavior of Zn ions and Co ions in the base catalysts was evaluated by ICP (Figure S6). The experimental data showed that pure ZIF-8 and ZIF-67 exhibited significant metal leaching in the PMS system. The Zn ion leaching concentration of ZIF-8 before PMS addition was 0.724 mg/L, and after PMS addition, they increased to 10.437 mg/L. The leaching concentration of Co ions from ZIF-67 increased from 1.409 mg/L to 11.048 mg/L. This could be ascribed to the susceptibility of the coordination bonds between the metal node (Zn/Co) and dimethylimidazole being easily cleaved under the acidic conditions of PMS, which led to the collapse of the framework and release of metal ions.^60^ In contrast, the leaching concentration of Zn ions from ZIF-8/AE-SiC decreased to 8.979 mg/L after the addition of PMS. This was due to the formation of Zn–O–Si covalent bonds between oxidized Si–O_*x*_ on the surface of AE-SiC and the metal nodes of ZIF-8, which enhanced the stability of the skeleton through chemical bonding. Moreover, the core–shell structure of ZIF-8@ZIF-67 further reduced the leaching concentrations of Zn and Co ions. For Zn ions, the leaching amounts before PMS addition were 0.109 mg/L and 5.679 mg/L after PMS addition. For Co ions, the leaching amounts before PMS addition were 0.616 mg/L and 5.345 mg/L after addition. This phenomenon was attributed to the ZIF-8 core providing mechanical support to ZIF-67, resulting in a synergistic stabilization effect.^29^ It was found that ZIF-8@ZIF-67/AE-SiC showed the best inhibition of metal leaching, with Zn ion/Co ion leaching concentrations of 0.178 mg/L for Zn and 0.195 mg/L for Co before PMS addition. After PMS addition, the leaching amounts were 1.285 mg/L for Zn and 0.932 mg/L for Co. The reason for the decrease in metal leaching was owing to the formation of strong Zn/Co–O–Si bonds between AE-SiC and ZIF-8@ZIF-67, while the shell–core structure of ZIF-8@ZIF-67 effectively prevented the large-scale disruption of the ZIFs structure.

To further evaluate the catalytic performance of ZIF-8@ZIF-67/AE-SiC, a comparison was conducted with other PMS activation catalysts reported for CIP degradation (Table S3). Compared with other reported catalysts, ZIF-8@ZIF-67/AE-SiC exhibited rapid and efficient CIP removal under a relatively low catalyst dosage and PMS dosage, highlighting its considerable potential for practical wastewater treatment applications.

### 3.5 Degradation Mechanism

The active species composition in the ZIF-8@ZIF-67/AE-SiC + PMS system was investigated using radical quenching experiments and EPR measurements. TBA was selected as the OH•-specific quenching agent, whereas EtOH was used as the quenching agent to eliminate OH• and SO_4_•–.^61^ In addition, l-his and PBQ were chosen as the quenching agents for single-linear oxygen (^1^O_2_) and superoxide radical (O_2_^•–^), respectively.^62,63^ The results are shown in Figure 7a, when equal amounts (100 mM) of TBA and EtOH are added, the corresponding CIP degradation rates are 92.98% and 21.21%, respectively. This indicated that TBA exerted only a minor inhibitory effect, whereas EtOH showed a markedly stronger suppression on CIP degradation. This suggested that SO_4_^•–^ and OH• initiated their actions simultaneously during PMS activation, but SO_4_^•–^ played a more critical role in the degradation process. When 1 mM *p*-benzoquinone was added, the CIP degradation rate plummeted to 45.52%, indicating that O_2_^•–^ played a certain role in the reaction. Furthermore, to investigate the effect of ^1^O_2_, 1 mM l-his was introduced. This resulted in the degradation efficiency of CIP decreasing to only 14.01% within 10 min, indicating that the inhibition effect of this scavenger was the most significant. Comprehensive analysis confirmed that ^1^O_2_ was the key species for CIP degradation in the ZIF-8@ZIF-67/AE-SiC+PMS system. ^1^O_2_ was usually generated by PMS self-decomposition reaction, as shown in eq 2, and the catalyst could accelerate the process to enhance the ^1^O_2_ yield. In summary, CIP degradation involved a synergistic mechanism of radicals and nonradicals, in which ^1^O_2_ played a dominant role through the nonradical pathway.^39^

2 HSO5−+SO5−→HSO4−+SO42‐+O21

**Figure 7:**
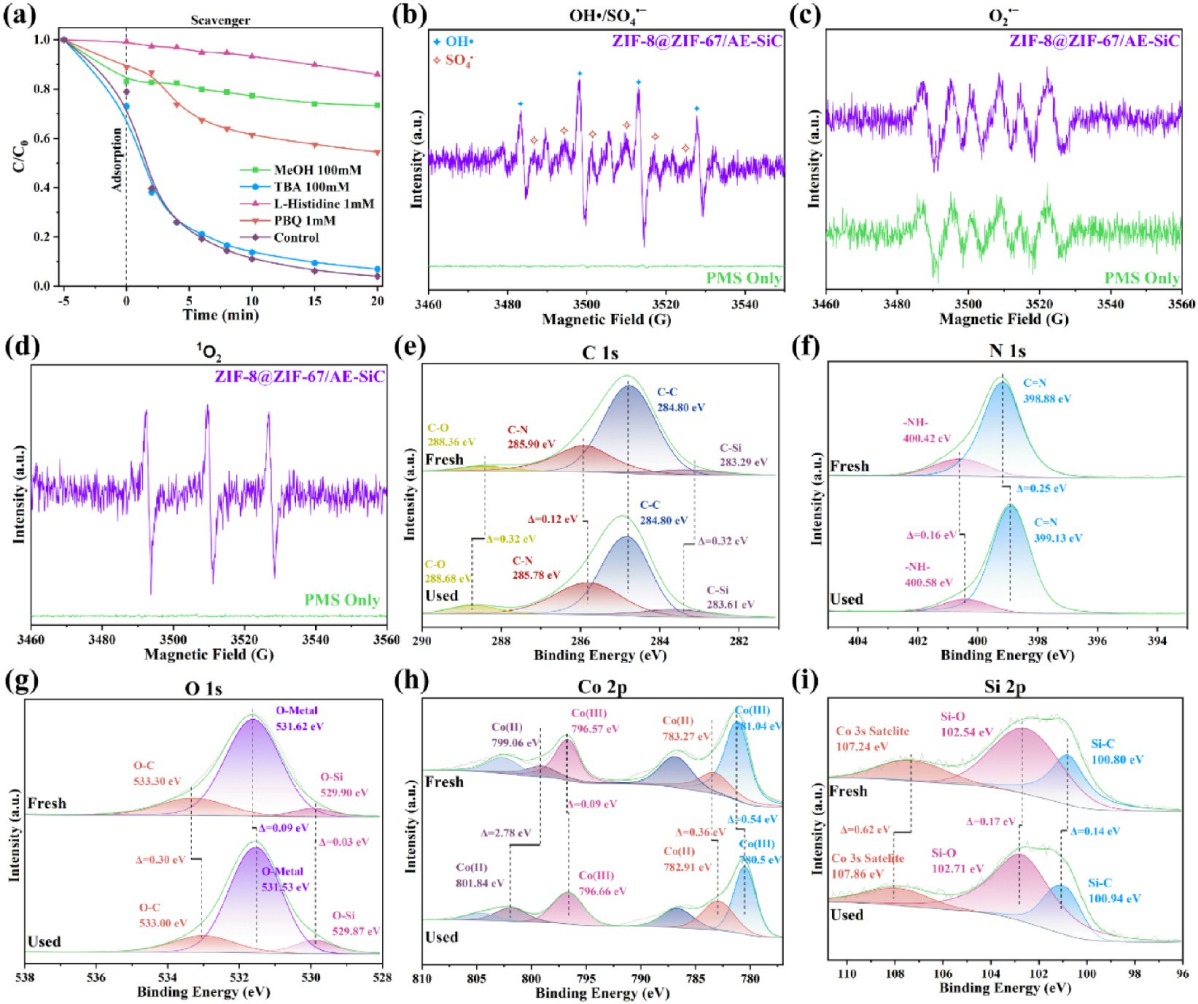
(a) Radical scavenging experiment, EPR spectra of (b–d) DMPO and (e) TEMP as the trapping agent in the ZIF-67/AE-SiC+PMS system. XPS spectra of (e) C 1s, (f) N 1s, (g) O 1s, (h) Co 2p, and (i) Si 2p.

The relative contributions of different reactive species during CIP degradation were estimated in Figure S7. Among the identified reactive species, ^1^O_2_ exhibited the highest contribution (95.54%), indicating that the degradation process predominantly proceeded through a nonradical oxidation pathway. Meanwhile, O_2_^•–^ and SO_4_^•–^ also played important roles, with contribution ratios of 80.96% and 73.34%, respectively, whereas OH• showed a comparatively limited contribution (18.94%). The substantial contribution of O_2_^•–^ may be associated with its participation in the generation of ^1^O_2_, thereby indirectly promoting pollutant degradation.^64^ It should be noted that different reactive oxygen species could transform into each other during PMS activation. Therefore, the calculated total contribution exceeded 100%.^65^

EPR was utilized to verify the results of the radical quenching experiment. As shown in Figure 7b, DMPO was used to detect OH• and SO_4_^•–^, and a very small OH• peak appeared when pure PMS was added. With the presence of ZIF-8@ZIF-67/AE-SiC, the DMPO–OH• adduct and DMPO–SO_4_^•–^ adduct showed the typical signals, indicating the presence of SO_4_^•–^ as well as OH• in the system, aligning with the results of quenching experiments with TBA and EtOH. When O_2_^•–^ was captured using DMPO in Figure 7c, the detection of characteristic quadruple peaks signal of the DMPO-O_2_^•–^ adduct confirmed the involvement of O_2_^•–^ during the reaction. However, the signal intensity similar to that of PMS, suggesting that the generation of O_2_^•–^ was mainly due to the self-generation of PMS rather than the catalyst-induced generation of PMS. The TEMP–^1^O_2_ adducts showed a distinct triple-linear signal in Figure 7d, which suggested that ZIF-8@ZIF-67/AE-SiC greatly promotes the generation of ^1^O_2_.

Comparison of the XPS survey spectra in Figure S8 showed that both fresh and post-reaction catalysts maintained the characteristic C 1s, N 1s, O 1s, Co 2p, Zn 2p, Si 2s, and Si 2p signal peaks. The Co 2p_3/2_ binding energy was significantly positively shifted by 1.61 eV, which was attributed to the redox reaction of Co ions with PMS during the activation of PMS.^66^ The C 1s spectrum in Figure 7e showed that the atomic percentages of C–C, C–N, and C–Si changed from 66.71%, 24.35%, and 5.51% to 55.13%, 31.36%, and 7.88%, respectively, indicating that there was no significant fracture of the carbon skeleton structure. The percentage of C–O increased from 3.43% to 5.63%, confirming that the CIP degradation intermediates were attached to the catalyst surface through hydrogen bonding and π–π stacking. The N 1s spectrum (Figure 7f) showed that the atomic percentages of the C=N bond and the liganded amino group changed from 88.83% and 11.17% to 80.99% and 19.01%, indicating that the imidazole ligand did not undergo significant denitrogenation, but some of the Co–N bonds might be protonated due to competitive adsorption of the intermediates. In the O 1s spectra (Figure 7g) showed that the percentages of O-Metal, O–Si, and O–C bonds varied from 78.07%, 5.23%, and 16.71% to 75.47%, 8.66%, and 15.87%, respectively. With a 3.43% increase in the O–Si bond and a shift in the binding energy of Δ = 0.03 eV, it was confirmed that the interfacial Si–O-M bond remained stable during the reaction. The Co 2p specta in Figure 7h demonstrate significant redox features. Co­(II) and Co­(III) shifted from 28.08% and 60.4% to 28.64% and 54.03% in atomic percentages, consistent with the redox pathway of Co^2+^/Co^3+^. The Si 2p spectra (Figure 7i) showed a significant redox feature of Si–C (100.8 eV) and Si–O (102.5 eV) bond shares, which shifted from 34.87% and 65.13% to 28.60% and 71.40%, confirming that the AE-SiC did not undergo a significant change. The binding energy shift (Δ = 0.62 eV) of the Co 3s satellite peak further verified that the redox cycle of Co dominated the catalytic process.

According to XPS results, the metal (M = Co, Zn) in ZIF-8@ZIF-67/AE-SiC could activate HSO_5_^–^ to generate SO_4_^•–^ with strong oxidizing ability, while M^n+^ was oxidized to M^(n+1)+^ (eq 3). Subsequently, M^(n+1)+^ further activated HSO_5_^–^ to generate SO_5_^•–^ and regenerated as M^n+^, forming an M^n+^-M^(n+1)+^–M^n+^ redox cycle (eq 4). Meanwhile, HSO_5_^–^ could react with water to ultimately generate O_2_^•–^ (eqs 5–8), and O_2_^•–^ could further react to form ^1^O_2_ (eq 9)­

3 $$M^{n+}{+\mathrm{HSO}}_{5}^{-}\to M^{\left(n+1\right)+}{+\mathrm{OH}}^{-}{+\mathrm{SO}}_{4}^{•-}$$

4 $$M^{\left(n+1\right)+}{+\mathrm{HSO}}_{5}^{-}\to M^{n+}{+H}^{+}{+\mathrm{SO}}_{5}^{•-}$$

5 $${\mathrm{HSO}}_{5}^{-}{+H}_{2}O\to H_{2}O_{2}{+\mathrm{HSO}}_{4}^{-}$$

6 $$H_{2}O_{2}\to {\mathrm{OH}}^{•}{+\mathrm{OH}}^{•}$$

7 $$H_{2}O_{2}{+\mathrm{OH}}^{•}\to H_{2}{O+\mathrm{HO}}_{2}^{•}$$

8 $${\mathrm{HO}}_{2}^{•}\to H^{+}{+O}_{2}^{•-}$$

9 O2•‐+HO2•→HO2+O21

Figure 8 showed the highest occupied molecular orbital (HOMO), lowest unoccupied molecular orbital (LUMO), surface electrostatic potential (ESP), and Hirshfeld’s charge of CIP were calculated by Gaussian 16W with Multiwfn 3.8. Figure 8a,b showed that the HOMO of CIP was mainly concentrated on the tetrahydropyridine ring, piperazine ring, and benzene ring, which were vulnerable to electrophilic attack and prone to losing electrons, while the LUMO was distributed on the quinolone ring, which was vulnerable to nucleophilic attack and tend to gain electrons. The ESP of the CIP molecule (Figure 8c) showed the difference in electron density, and the red negative potential region near the carboxyl group of the CIP molecule may be combined with the negative potential of the structure of the ZIFs, which provided a favorable condition for the formation of hydrogen bonds. Meanwhile, the π–π stacking interaction improved the adsorption stability. The inhomogeneity of ESP distribution led to charge redistribution, which contributed to the adsorption of CIP molecules. Based on Fukui function theory, regions of high values of attack index predicted that CIP molecules were more susceptible to specific ROS attack.^67^ As shown in Figure 8d, N15 (*f* ^–^ = 0.104), C10 (*f* ^–^ = 0.0689), and O23 (*f* ^–^ = 0.0606) had the highest *f* ^–^ values, indicating that these sites were more susceptible to electrophilic attack (e.g., ^1^O_2_). The higher *f* ^+^ values of C4 (*f* ^+^ = 0.1243), O23 (*f* ^+^ = 0.0802), and O1 (*f* ^+^ = 0.0573) in Figure 8e showed their susceptibility to nucleophilic attack. The highest *f* ^0^ values of C4 (*f* ^0^ = 0.0712), O23 (*f* ^0^ = 0.0704), and N15 (*f* ^0^ = 0.0601) in Figure 8f indicated that these sites were susceptible to OH• attack. Combining the electrophilic/nucleophilic reactivity, the condensed dual descriptor (CDD) Δ*f* values are shown in Figure 8g, C4 (Δ*f* = 0.1062), C22 (Δ*f* = 0.0356), and H25 (Δ*f* = 0.0315) had higher values, indicating their outstanding reactivity with ^1^O_2_

**Figure 8:**
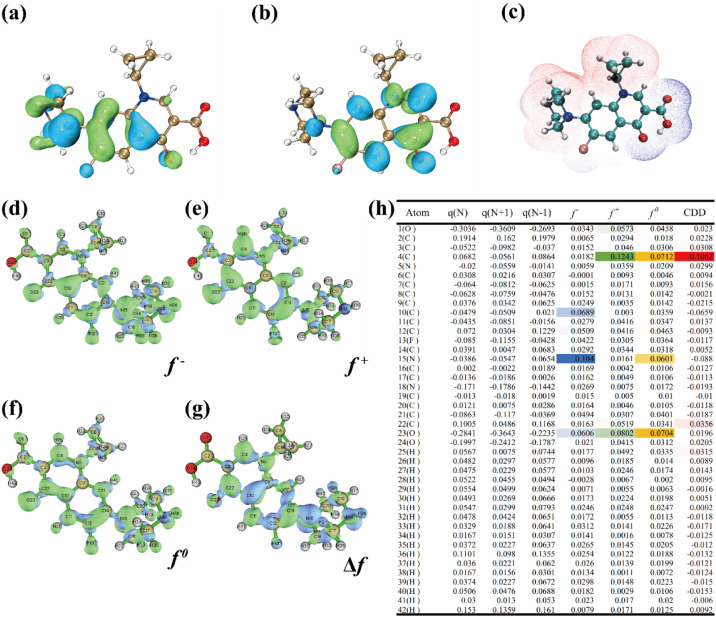
(a) HOMO, (b) LUMO, (c) ESP, and (d) *f* ^–^, (e) *f^+^*, (f)*f* ^0^, (g) Δ*f*, and (h) Hirshfeld charges of CIP.

As shown in Figure S9, the TOC removal efficiencies of CIP reached 21% and 32% after 10 and 20 min of reaction, respectively. These results indicate that the ZIF-8@ZIF-67/AE-SiC+PMS system possesses a certain mineralization capability toward CIP. However, incomplete mineralization was still observed, suggesting the presence of partially oxidized intermediate products in the reaction system. Further, possible degradation pathways of CIP in the ZIF-8@ZIF-67/AE-SiC+PMS system were identified, as shown in Figure 9. In pathway 1, the fluorine atom in the piperazine ring generated intraring angular tension through an induced effect, which prompts OH• to attack the carbon atoms of the piperazine ring, leading to partial breakage of the piperazine moiety and the formation of the intermediate P1 (*m*/*z* = 362). P1 was followed by the partial elimination of formaldehyde from the amide portion after the further defluorination of P1 to generate P2 (*m*/*z* = 288), which was oxidatively stripped of its diethylamine form with NO_2_ to generate P3. In path 2, the piperazine ring of CIP underwent an electrophilic reaction, which allowed the hydrogen abstraction reaction due to SO_4_^•–^ attack to form P4. P4 was subsequently oxidized to P5 (*m*/*z* = 348) by C–N bond breaking, and the loss of amide by P5 underwent further electrophilic reaction to generate P6 (*m*/*z* = 366). Further, P6 was subsequently obtained under the continuous attack of radicals by removing the aldehyde group to obtain the intermediate P7 (*m*/*z* = 291), and then finally C–N bond breaking occurred to obtain the product P8 (*m*/*z* = 263), which could be generated as P3 by the reaction of defluorination and C–N bond cleavage on the benzene ring of P8. In pathway 3, the defluorination of the quinolone in the CIP generated P9 (*m*/*z* = 330), which can be converted to P10 (*m*/*z* = 285) through the breakage of the carboxyl group. The piperazine ring of P10 underwent oxidation to generate P11 (*m*/*z* = 304). The piperazine ring of P11 underwent breakage to form P12 (*m*/*z* = 274). Finally, P3, P8, and P12 underwent a ring-opening reaction, which ultimately reformed products such as carbon dioxide and water. The ecotoxicity of CIP and the degradation intermediates were analyzed by Toxicity Evaluation Software (T.E.S.T.) (Figure S10). The 48 h LC50 of CIP for *Daphnia magna* was 2.84 mg/L, which was regarded as toxic. The LC50 values of the degradation products P3, P7, P1, P9, P12, and P6 were elevated, and the acute toxicity was significantly reduced. The predicted bioconcentration factor (BCF) of CIP was 13.21, and the BCFs of all intermediates were reduced except for P10 and P11. In the developmental toxicity assessment, CIP had a developmental toxicity index of 1.09 and was classified as developmentally toxic, whereas the developmental toxicity of most intermediates was markedly reduced. Mutagenicity tests showed that CIP and its degradation products P4, P11, P1, P9, and P10 were mutagenic, while the other intermediates were non-mutagenic. This indicated that the ZIF-8@ZIF-67/AE-SiC+PMS system can effectively degrade CIP and reduce its toxicity.

**Figure 9:**
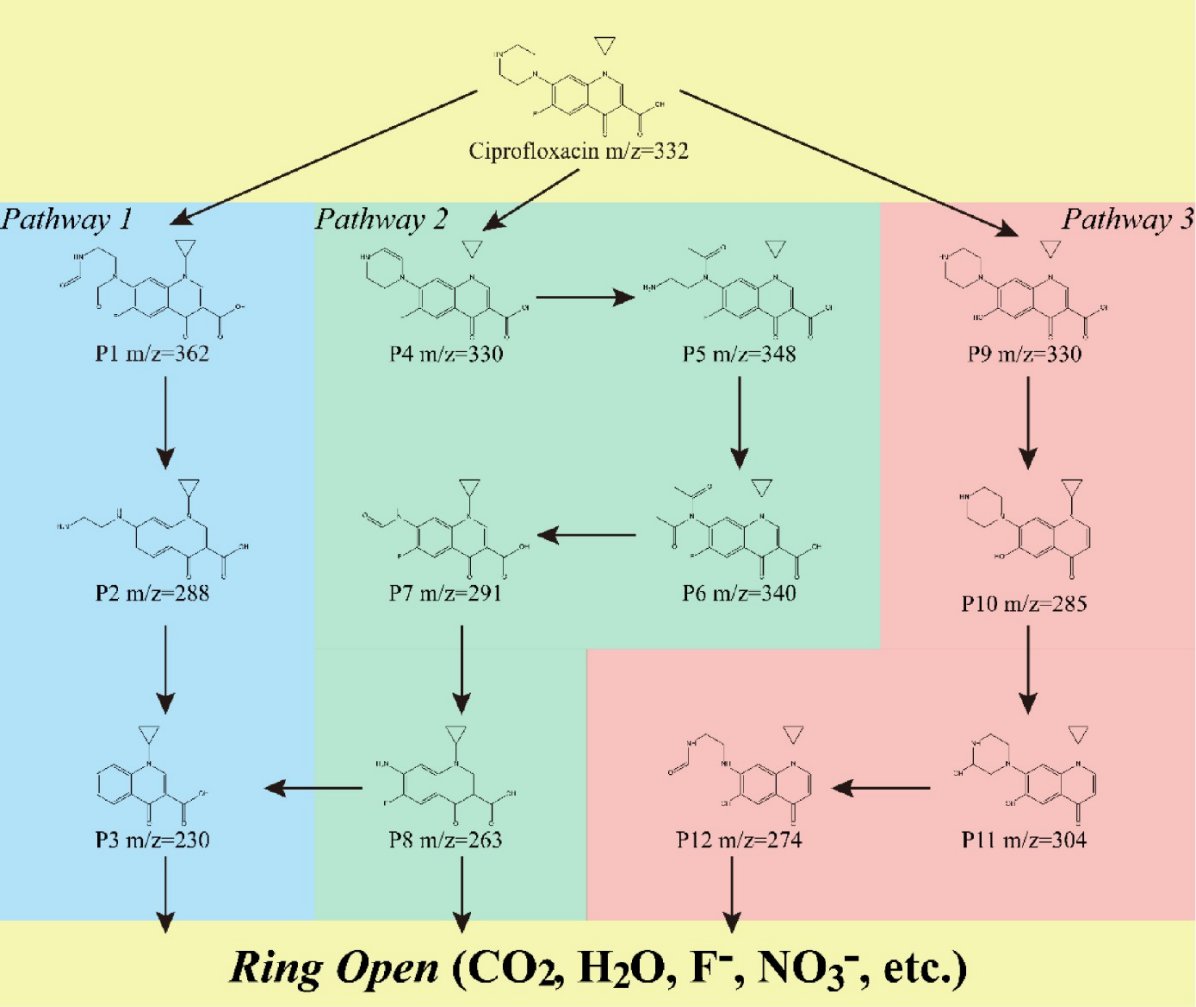
Proposed pathways of CIP degradation.

Based on the above, the adsorption mechanism of Sr and the degradation mechanism of CIP by ZIF-8@ZIF-67/AE-SiC are shown in Figure 10. For adsorption, based on the adsorption kinetics and adsorption isotherm simulation results, the adsorption process of Sr^2+^ by ZIF-8@ZIF-67/AE-SiC was mainly dominated by physical adsorption and multilayer adsorption. According to the zeta potential data, the surface of ZIF-8@ZIF-67/AE-SiC carried a negative charge under neutral conditions and can bind with positively charged Sr^2+^ ions through electrostatic interaction, thereby achieving effective adsorption of Sr. For degradation, based on the EPR, radical quenching experiments, and XPS analysis, Co and Zn acted as the reactive metal active sites, activating PMS through electron transfer to generate SO_4_^•–^, OH•, and ^1^O_2_ as the primary active species. These species degraded organic pollutants into low-toxicity or nontoxic intermediate products, thereby achieving effective degradation of CIP. By constructing core–shell interfacial composite materials, the synergistic removal of radionuclides and organic pollutants was achieved. The material demonstrated excellent stability and high activity even under complex environmental conditions, providing a new strategy for the development of multifunctional water treatment materials and showing great potential for applications in environmental remediation and sustainable water resource utilization.

**Figure 10:**
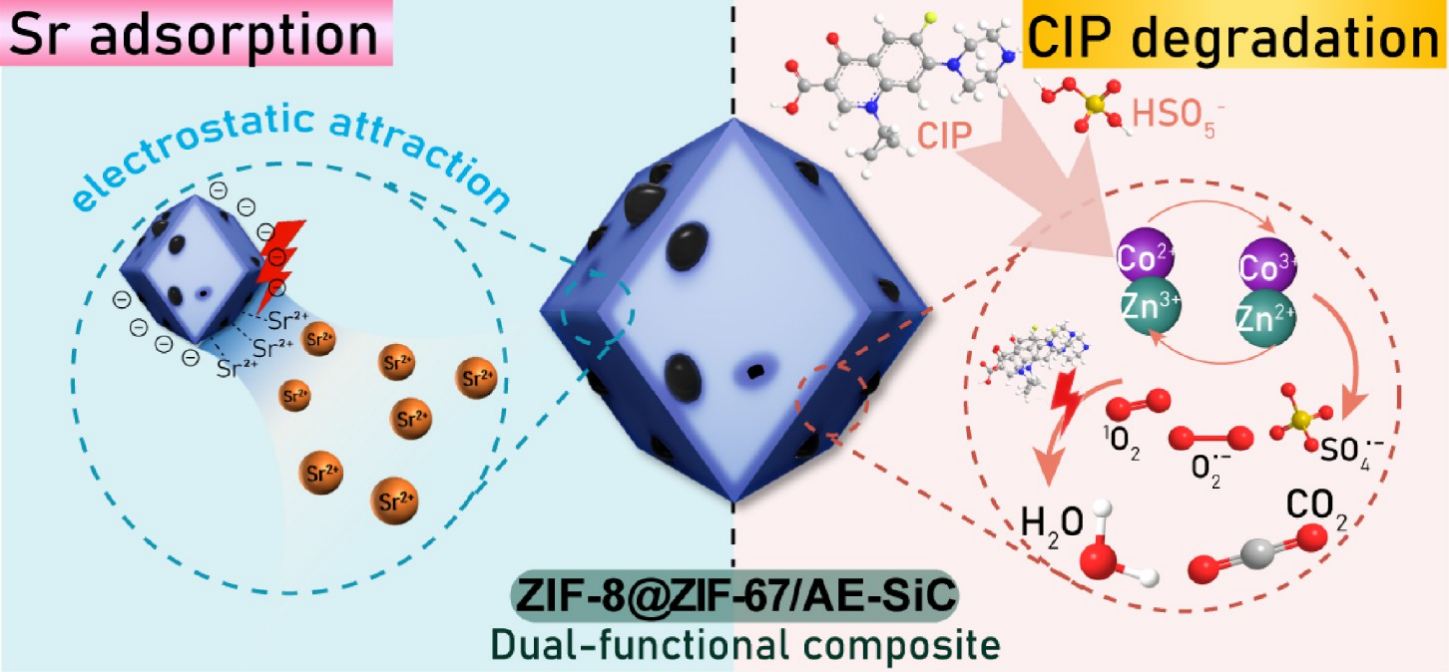
Schematic diagram of Sr adsorption and CIP degradation by ZIF-8@ZIF-67/AE-SiC.

## 4 Conclusion

In this work, the prepared bifunctional core–shell structured composite material ZIF-8@ZIF-67/AE-SiC exhibited dual functionality for radionuclide adsorption and antibiotic degradation, achieving a Sr^2+^ adsorption capacity of 50.3 mg/g and >95% CIP removal within 20 min, with broad-spectrum reactivity against other pollutants. The composite maintained high activity across a wide pH and temperature range and in complex ionic environments, while reducing Zn/Co leaching by 60–80%, thereby ensuring superior structural stability and reusability. The adsorption process conformed to the pseudo-first-order kinetic model and the Freundlich isotherm, indicating that physical adsorption and multilayer adsorption were dominant mechanisms. Degradation mechanistic investigations revealed that in the ZIF-8@ZIF-67/AE-SiC system, SO_4_^•–^ and ^1^O_2_ were identified as the key active species responsible for the CIP degradation. Potential reaction sites for CIP and three degradation pathways were proposed, and a predictive analysis of the toxicity of degradation products was conducted. The constructed core–shell structure and Si–O-M bonds enable electronic regulation and enhanced stability of the composite material, achieving reversible Co^2+^/Co^3+^ cycling. This work offers a strategic approach for coremediation of radionuclides and organic pollutants in realistic wastewater systems and holds promise for the synergistic treatment of multiple pollutants.

## Supplementary Material



## Notes

Chemicals and reagents, detailed synthesis procedures, adsorption and degradation experiments, XRD, pollutant degradation performance, degradation cycle tests and characterization, ion leaching, ROS contribution, XPS survey, TOC, toxicity tests, and comparison tables of Sr^2+^ adsorption and catalytic degradation performance (PDF)
