# Supplementary material for: Multifunctional Metal–Organic Framework/Alkali-Etched Silicon Carbide Composite for Efficient Strontium Adsorption and Ciprofloxacin Removal
Source: ACS Appl Mater Interfaces. 2026 Jul 13;18(28):38882–98. doi: 10.1021/acsami.6c08717 (PMC13397489; doi:10.1021/acsami.6c08717)
Supplement: Supplementary file 1 [file am6c08717_si_001.pdf]

Supporting Information

Multifunctional Metal-Organic Framework/ Alkali-etched Silicon Carbide Composite for Efficient Strontium Adsorption and Ciprofloxacin Removal

Yongxin Lei[a‡], Zhencong Liu[a‡], Wenjie Qin[a], Zihan Li[a], Dongci Wei[a], Santosh K. Tiwari[c], Xinpeng Wang[a], Yanqiu Zhu[a,d]*, Nannan Wang[a]*, Haiyan Mou[a,b]*.

[a] State Key Laboratory of Featured Metal Materials and Life-cycle Safety for Composite Structures, MOE Key Laboratory of New Processing Technology for Nonferrous Metals and Materials, School of Resources, Environment and Materials, Guangxi University, Nanning, 530004, China

[b] Institute for Disaster Management and Reconstruction, Sichuan University-The Hong Kong Polytechnic University, Chengdu 610065, China

[c] Centre for New Materials & Surface Engineering, Department of Chemistry, NMAM Institute of Technology, Nitte University, Karnataka, 574110, India

[d] Faculty of Environment, Science and Economy, University of Exeter, EX4 4QF, UK

[‡] These authors contributed equally.

*Corresponding author. **Yanqiu Zhu:** E-mail: *Y.Zhu@exeter.ac.uk* ORCID: *0000-0003-3659-5643*; **Nannan Wang:** E-mail: *wangnannan@gxu.edu.cn* ORCID: *0000-0002-9013-0612*; **Haiyan Mou:** E-mail: *mouhaiyan@scu.edu.cn* ORCID*: 0009-0006-2887-4158*

Text S1. Chemicals

2-Methylimidazole (2-Melm, 99.0%), Cobaltous nitrate hexahydrate ($Co(NO_3)_2$• $6H_2O$, 99.0%), Strontium nitrate ($Sr(NO_3)_2$, 99%), Potassium peroxymonosulfate (PMS, $KHSO_5$•$0.5KHSO_4$•$0.5K_2SO_4$, 42%~46% $KHSO_5$ basis), Ciprofloxacin hydrochloride (CIP, 98%), 1,4-Benzoquinone (PBQ, 99%) were acquired from Maclin Biochemical Technology Co., Ltd. (Shanghai, China). Tetracycline hydrochloride (TCH, 96%) was acquired from Aladdin Biochemical Technology Co., Ltd. (Shanghai, China). Methanol (MeOH, 99.9%), NaCl (99%), and $NaHCO_3$ (99%) were acquired from Guangdong Guanghua Sci-Tech Co., Ltd. (Guangzhou, China). NaH_2PO_4 (99%), and L-histidine (99%) were acquired from Sinopharm Chemical Reagent Co., Ltd.(Shanghai, China). Tert-butyl alcohol (TBA, 99.5%), and Rhodamine B (RhB, AR) were acquired from Tianjin Damao Chemical Reagent Factory (Tianjin, China). KNO_3 (99%) was acquired from Chengdu Jinshan Chemical Reagent Co., Ltd. (Chengdu, China). β-SiC NPs 50nm were acquired from Shanghai MCC New Materials Co., Ltd. (Shanghai, China).

Text S2. Preparation of ZIF-8, ZIF-67 and ZIF-8@ZIF-67

Preparation of ZIF-8: Place 1.785 g (6 mmol) of zinc nitrate hexahydrate into 60 mL of methanol solution, referred to as Solution A. Place 1.970 g (24 mmol) of dimethylimidazole into 60 mL of methanol solution, referred to as Solution B. After stirring Solution A and Solution B separately for 30 minutes, slowly add Solution B to Solution A. Stir the mixture at room temperature for 24 h. Centrifuge the resulting grayish-white precipitate, wash it three times with methanol, and finally vacuum-dry it at 60℃ to obtain pure ZIF-8.

Preparation of ZIF-67: Place 1.746 g (6 mmol) of cobalt nitrate hexahydrate into 60 mL of methanol solution; this solution is designated as Solution A. Place 1.970 g (24 mmol) of dimethylimidazole into 60 mL of methanol solution; this solution is designated as Solution B. After stirring Solution A and Solution B separately for 30 minutes, slowly add Solution B to Solution A. Stir the combined solution at room

temperature for 24 h. Centrifuge the resulting purple precipitate, wash it three times with methanol, and finally vacuum-dry it at 60℃ to obtain pure ZIF-67.

Preparation of ZIF-8@ZIF-67: Disperse all the ZIF-8 prepared in the previous section into 30 mL of methanol to form a mixed solution, designated as Solution A. Place 1.746 g (6 mmol) of cobalt nitrate hexahydrate into 60 mL of methanol solution, designated as Solution B. Place 1.970 g (24 mmol) of dimethylimidazole into 60 mL of methanol solution, designated as Solution C. Slowly add Solution B to Solution A while stirring for 30 minutes. Subsequently, solution C was added to the above mixture. After stirring the mixture at room temperature for 24 h, the resulting light purple precipitate was separated by centrifugation, washed three times with methanol, and vacuum-dried at 60 ℃ to obtain ZIF-67@ZIF-8.

Text S3. Adsorption experiments

Prepare a 40 mg/L Sr^{2+} solution and adjusting the pH. Experimental batches are conducted in centrifuge tubes containing a certain amount of adsorbent and Sr^{2+} solution of prescribed concentration, using an air bath oscillator stirred at 200 rpm. Filter the adsorbed solution using a 0.22 μm pore size injection filter equipped with a hydrophilic membrane. The concentrations were determined using AAS, and the reported results represent the average of three replicates. The removal efficiency of Sr^{2+} substances E(%) (**Eq. (1)**), equilibrium adsorption capacity q_e (mg/g) (**Eq. (2)**) and distribution coefficient K_d (mL/g) (**Eq. (3)**) can be calculated:

$$E\% = \frac{C_0\text{-}C_e}{C_0} \times 100\% \quad \textbf{(1)}$$

$$q_e = \frac{V}{m}(C_0\text{-}C_e) \quad \textbf{(2)}$$

$$K_d = \frac{1000V}{m}\frac{(C_0\text{-}C_e)}{C_e} \quad \textbf{(3)}$$

Where, C_0 and C_e represent the initial and equilibrium concentrations of Sr^{2+} respectively, V (mL) is the volume of the solution and m (mg) is the mass of the adsorbent.

3.1 Effect of the initial pH

Adjust the pH of the Sr^{2+} solution (40 mg/L) using HCl and NaOH solutions to conduct adsorption experiments within a pH range of 4 to 10. The residual concentration of Sr^{2+} after adsorption was measured by AAS and the optimum pH was determined by calculating the adsorption capacity.

3.2 Adsorption kinetics experiment

The adsorption kinetic behavior of ZIF-8@ZIF-67/AE-SiC composite aerogel towards Sr^{2+} was investigated using a time-sequence adsorption experiment: the adsorbent was placed in a Sr^{2+} solution with an initial concentration of 40 mg/L, and dynamic adsorption was conducted under room temperature conditions for 0-120 min. To systematically reveal the rate-controlling steps of the adsorbent during the adsorption process and the diffusion mechanism of Sr^{2+} within its interior, the experimental data were fitted using the pseudo-first-order model (**Eq. (4)**), pseudo-second-order model (**Eq. (5)**). The pseudo-first-order kinetic model is primarily used to describe physical adsorption mechanisms dominated by diffusion processes; the pseudo-second-order model effectively distinguishes the dominance of chemical adsorption (such as ion exchange or surface complexation).

$$q_t = q_e(1 - e^{-k_1 t}) \tag{4}$$

$$q_t = \frac{q_e^2 k_2 t}{1 + q_e k_2 t} \tag{5}$$

In the equation, q_t and q_e represent the adsorption capacity at any time t (min) and equilibrium (mg/g) respectively, k_1 and k_2 represent the rate constants of the quasi first-order kinetic model and the quasi second-order kinetic model respectively.

3.3 Adsorption isotherm experiment

To clarify the adsorption mechanism and site characteristics for ZIF-8@ZIF-67/AE-SiC composite aerogel for Sr^{2+}, isothermal adsorption experiments were carried out under thermodynamic equilibrium conditions: the adsorbent was placed in a single Sr^{2+} system (concentration gradient 10-700 mg/L) and vibrated at 200 rpm for 60 min. Langmuir (**Eq. (6)**), assuming single-layer chemisorption and homogeneous adsorption sites), Freundlich (**Eq. (7)**), assuming multi-layer adsorption and surface heterogeneity.

$$q_e = q_m \frac{K_L C_e}{1+ K_L C_e} \tag{6}$$

$$q_e = K_F C_e^{1/n} \tag{7}$$

$$R_L = \frac{1}{1+ K_L C_0} \tag{8}$$

q_e and q_m are equilibrium adsorption capacity (mg/g) and theoretical adsorption capacity (mg/g) respectively. K_L is the Langmuir constant characterizing the adsorption energy. K_F is the Freundlich constant related to the interaction energy. Parameter n represents Freundlich index, reflecting the adsorption strength. When n>1, the adsorption of the target adsorbate is a favorable process. R_L (**Eq. (8)**) is the Langmuir model separation factor parameter and C_0 (mg/L) represents the initial concentration of Sr^{2+}.

3.4 Competitive adsorption

To investigate the practical application potential of ZIF-8@ZIF-67/AE-SiC composite aerogel in complex aqueous environments, multi-ion competitive adsorption experimental system was designed: Simulated competitive system: The mixed solution containing Ni^{+}, Mn^{2+}, Na^{+}, K^{+}, Sr^{2+}, and Cs^{+} (each ion concentration at 1 mmol/L) was prepared. After constant-temperature shaking adsorption (RT, 1h), the adsorption amounts of each ion were determined by ICP-MS.

Text S4. Degradation experiment

The degradation experiments of CIP were conducted in a 250 mL beaker containing 100 mL of contaminated solution, which was prepared by adding CIP powder to deionized water, resulting in an initial CIP concentration of 20 mg/L. A 5 mg dose of catalyst was added to the solution, which had a pH value of 6.8, and the mixture was sonicated for 60 seconds. Subsequently, 0.0614 g (1 mM) of PMS was introduced, and the solution was stirred for 10 minutes at 350 r/min.

At 0, 2, 4, 6, 8, and 10 minutes, 4 mL samples were taken and filtered through a 0.22 μm syringe filter membrane. The concentration of CIP was measured using a UV-Vis spectrophotometer at approximately 276 nm. Unless otherwise specified, the catalyst dosage, pH value, PMS dosage, and pollutant concentration were kept constant

under the above conditions. In **Eq. (9)**, η (%) represents the removal efficiency, while C_t (mg/L) and C_0 (mg/L) correspond to the CIP concentration at time t and the initial concentration of CBZ in the solution, respectively. In **Eq. (10)**, k_{obs}(t) represents the pseudo-first-order reaction rate constant, and t (min) refers to the reaction time.

$$\eta = \frac{C_0 - C_t}{C_0} \times 100\% \quad \textbf{(9)}$$

$$\ln \frac{C_t}{C_0} = -k_{obs} t \quad \textbf{(10)}$$

To predict reaction sites, the molecular structure of CIP was constructed using GaussView 6.0 and optimized using Gaussian 16 software at the B3LYP/6-311G (d) level. Subsequently, based on Hirshfeld charges, the Fukui function of CIP molecules was calculated using Multiwfn 3.6 software, and the energy values of the lowest unoccupied molecular orbital (LUMO) and the highest occupied molecular orbital (HOMO) were obtained, thereby determining the most reactive sites in the molecule. All calculation results are visualized using VMD 1.9.3 software.

Text S5. Characterizations

X-ray diffraction (XRD) was performed using Bruker XRD angle measuring instrument D8 Discover X-ray diffractometer (Bruker AXS Karlsruhe, Germany) and SmartLab 3 kW X-ray diffractometer (Nippon Rikki, Japan). The radiation source is Cu K α radiation, and all samples are tested under 40 kV and 30 mA conditions, scanning at a rate of 10° per minute in the range of 5° to 80°. Fourier Transform Infrared Spectroscopy (FT-IR) is an effective characterization method for testing the surface functional group information of prepared catalysts. Using the Fourier transform infrared spectrometer (Thermo Fisher Scientific Nicolet iS50, USA), the spectra of the prepared samples were collected within the testing range of 4000-400 cm^{-1}. The scanning electron microscopy (SEM) technique and its combined energy dispersive spectroscopy (EDS) system were used to comprehensively characterize the microstructure, elemental composition, and distribution characteristics of the catalyst(Sigma 300, Carl Zeiss, Germany). Atomic scale characterization of sample nanostructures was performed using transmission electron microscopy (TEM) and its

combined energy dispersive spectroscopy (EDS) system (Tecnai F20, FEI Corporation, USA). The surface chemical composition and electronic structure of the catalyst were deeply analyzed using X-ray photoelectron spectroscopy (XPS) technology (ESCALAB 250Xi, Thermo Scientific, USA). The specific surface area of the material was studied using N_2 adsorption desorption isotherms by fully automatic specific surface area analyzer (TriStar II 3020, USA). The adsorption data of the samples was obtained using the atomic absorption spectrophotometer (SHIMADZU AA-7000, Japan) to analyze their adsorption performance. The Zeta potential characterization was carried out using the NanoBrook Omni multifunctional potential analysis system (Brookhaven Instruments, USA), equipped with a phase analysis light scattering (PALS) detection module. The Zeta potential of the sample was measured by dynamic light scattering (DLS) under constant temperature conditions of 25 ± 0.1 ℃. The Lambda 365 UV visible spectrophotometer system produced (PerkinElmer, Germany) is used for both quantitative analysis of reactant concentration and testing of UV Vis diffuse reflectance spectroscopy (DRS) solid sample UV absorption performance. Mass spectrometry data acquisition was performed using a Q-Exactive Orbitrap high-resolution mass spectrometer. Quantitative detection of metal ion leaching concentration in the reaction solution was performed using an inductively coupled plasma spectroscopy system (Thermo Fisher Scientific iCP 7000, USA). Electron paramagnetic resonance spectroscopy analysis was performed on an EMXplus-6/1 X-band (9.85 GHz) paramagnetic resonance spectrometer (AXS Bruker in Karlsruhe, Germany).

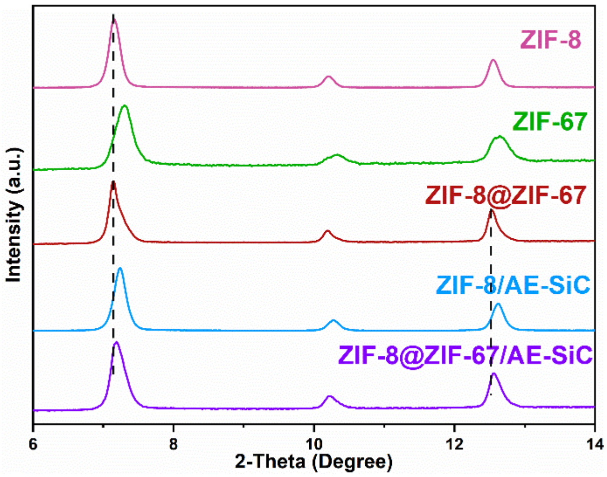

Figure S1 XRD magnified images of ZIF-8, ZIF-67, AE-SiC, ZIF-8@ZIF-67, ZIF-8/AE-SiC, and ZIF-8@ZIF-67/AE-SiC in the ranges of 2θ = 6°–14°.

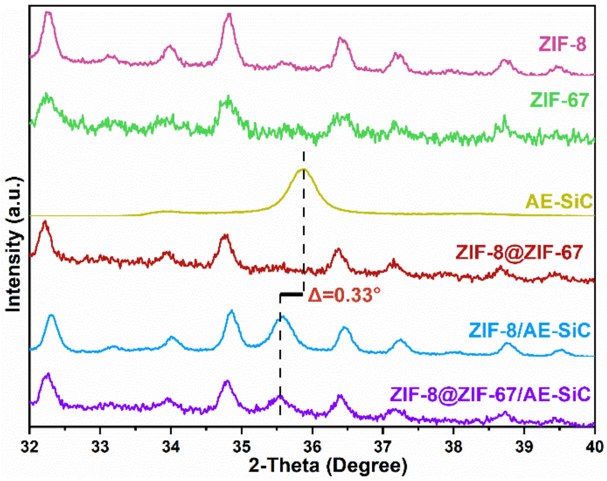

Figure S2 XRD magnified images of ZIF-8, ZIF-67, AE-SiC, ZIF-8@ZIF-67, ZIF-8/AE-SiC, and ZIF-8@ZIF-67/AE-SiC in the ranges of 2θ = 32°–40°.

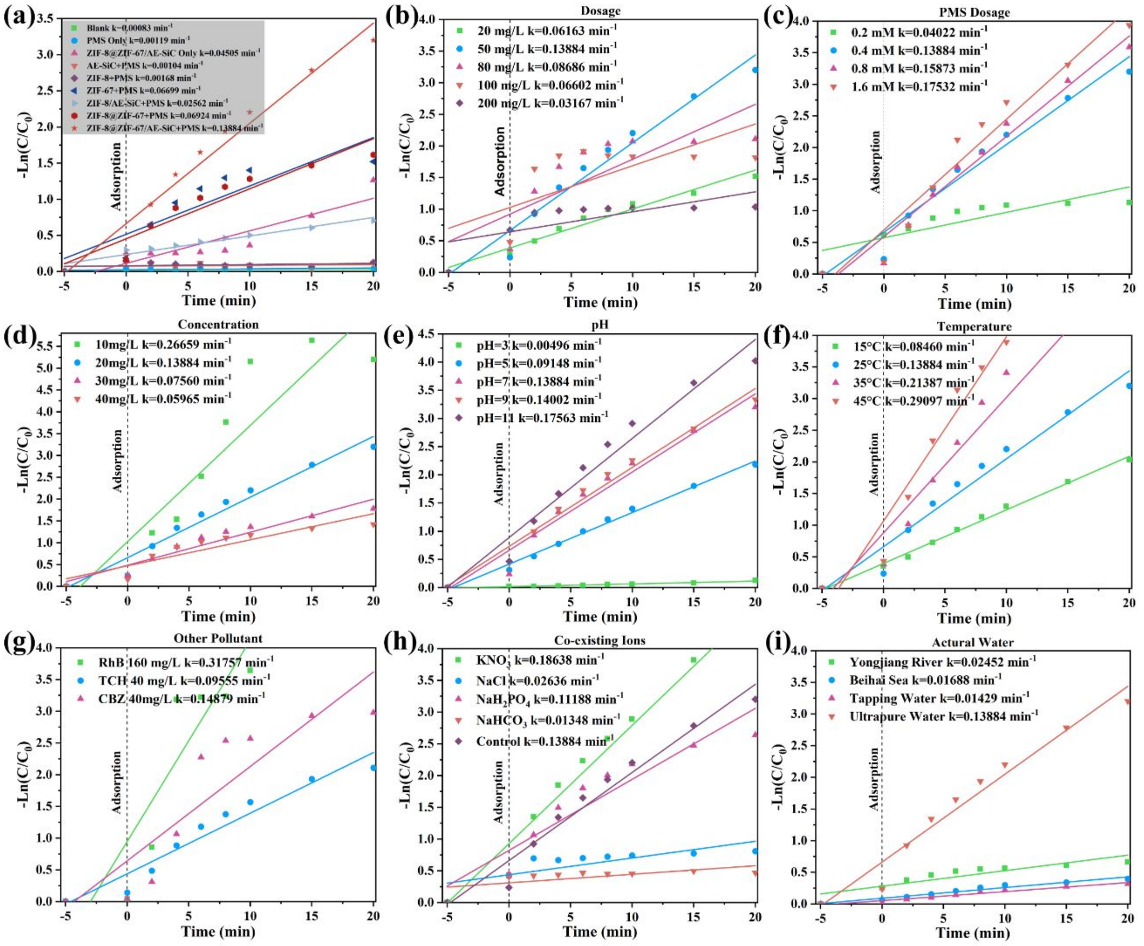

Figure S3 k_{obs} of (a) different catalysts, (b) catalyst dosage, (c) PMS dosage, (d) CIP concentration, (e) initial pH, (f) temperature on CIP removal, (g) other pollutants, (h) co-existing ions and (i) different water matrices in the ZIF-8@ZIF-67/AE-SiC+PMS system.

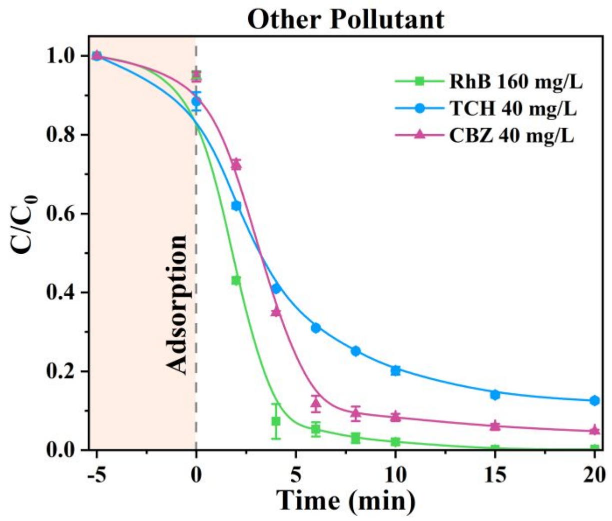

Figure S4 The ZIF-8@ZIF-67/AE-SiC+PMS system's degradation capacity for different pollutants.

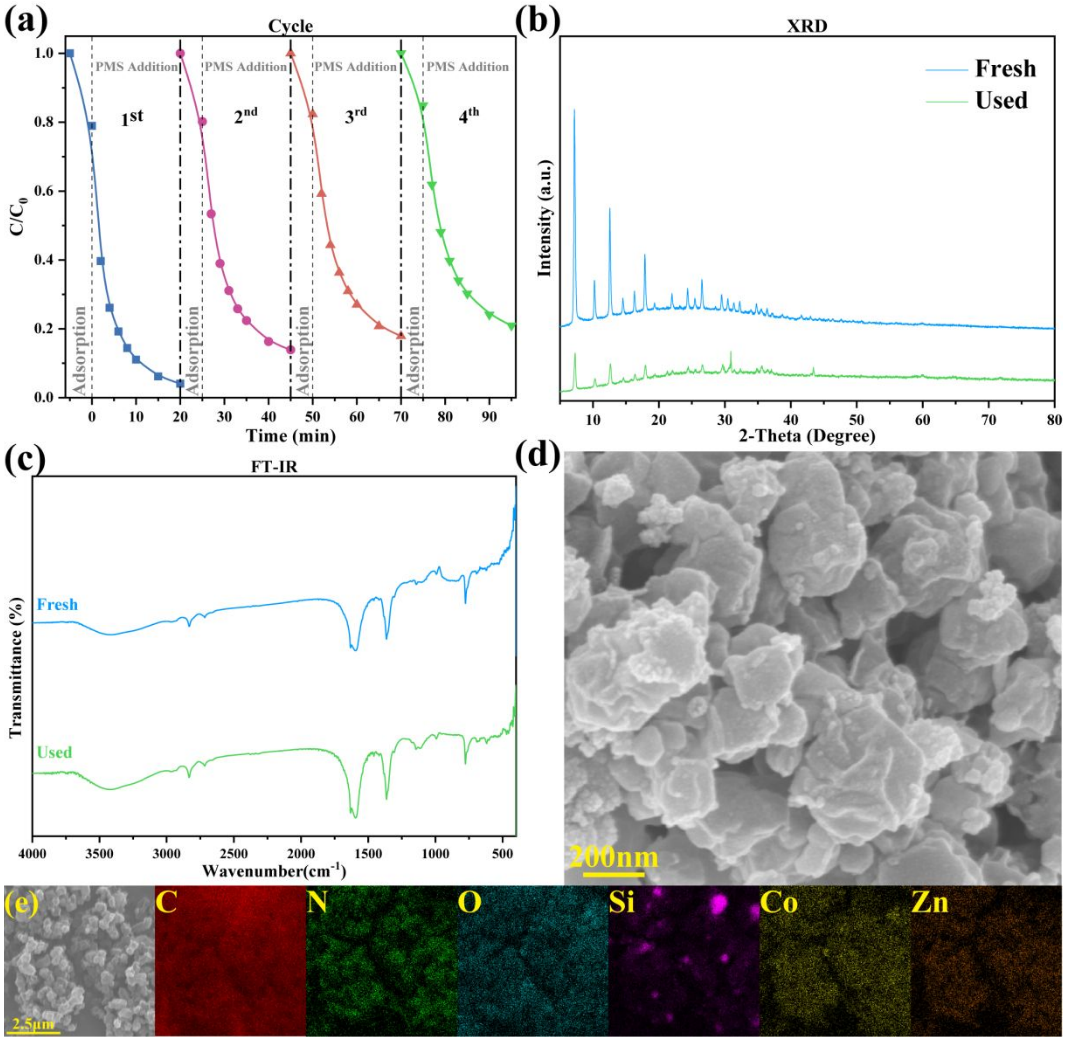

Figure S5 (a) Cycle test of ZIF-67/AE-SiC. (b) XRD patterns, (c) FT-IR spectra of ZIF-8@ZIF-67/AE-SiC comparison between fresh and used. (d) SEM images and (e) SEM-EDS mapping of used ZIF-8@ZIF-67/AE-SiC.

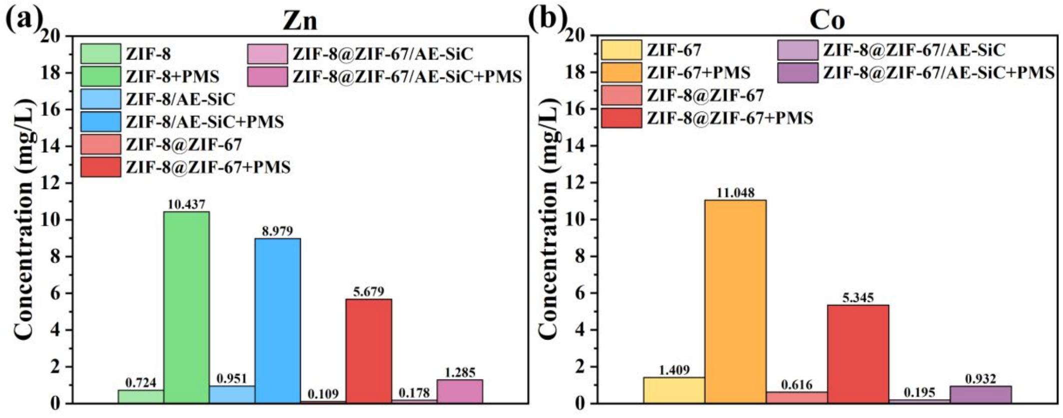

Figure S6 (a) Zn and (b) Co ion leaching tests of ZIF-8, ZIF-67, ZIF-8/AE-SiC, ZIF-8@ZIF-67 and ZIF-8@ZIF-67/AE-SiC with and without the addition of PMS.

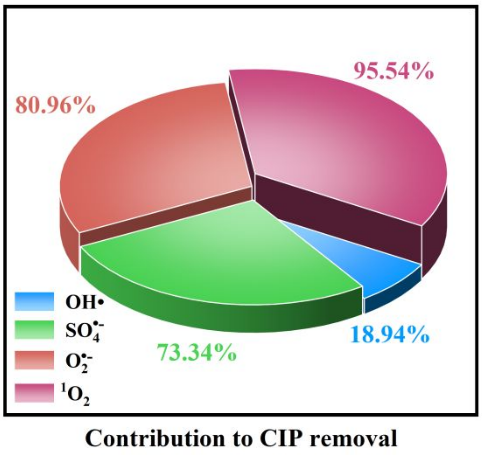

Figure S7 Calculate the contribution rate of different active species

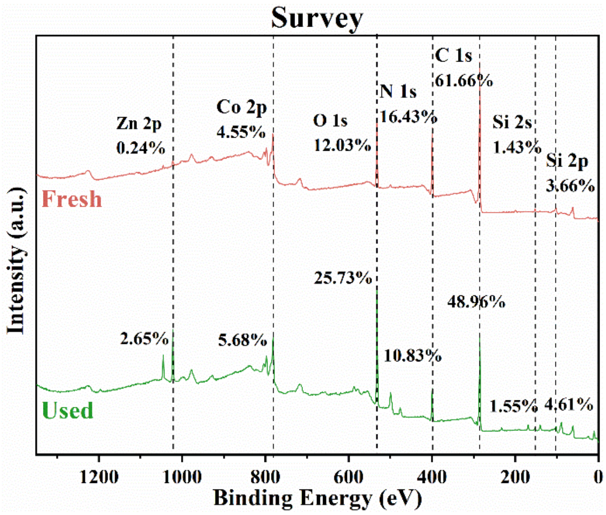

Figure S8 XPS survey of ZIF-8@ZIF-67/AE-SiC.

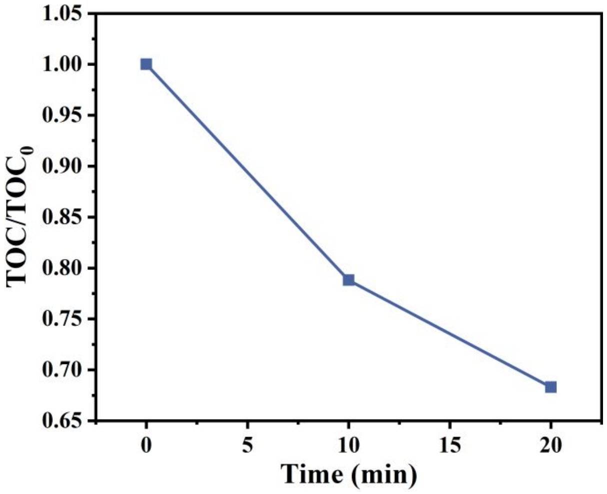

Figure S9 TOC analysis in ZIF-8@ZIF-67/AE-SiC+PMS systems

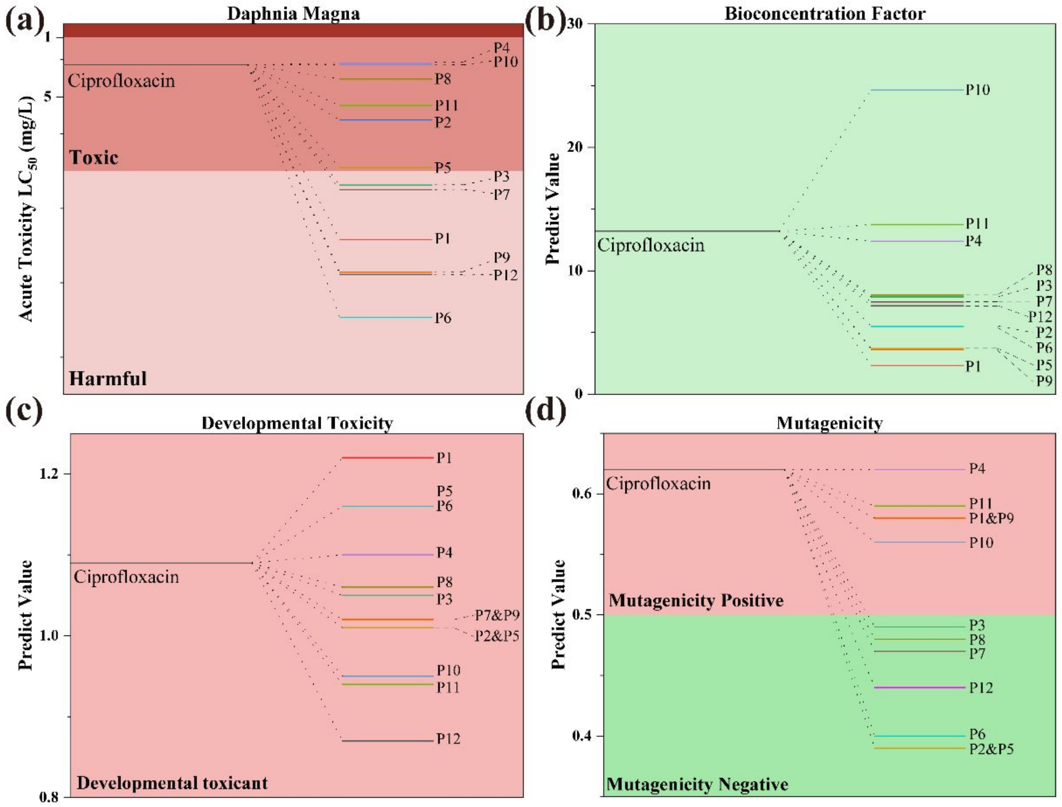

Figure S10 The calculated toxicity for (a) LC50 for daphnia magna, (b) bioconcentration factor, (c) developmental toxicity, and (d) mutagenicity of CIP and its degradation intermediates within the degradation pathways in the ZIF-8@ZIF-67/AE-SiC+PMS system.

Table S1. Parameters of the Sr dimensionless separation factor (R_L) of ZIF-8@ZIF-67/AE-SiC.

Adsorbent	Initial concentration (mg/L)									
	0	5	10	20	40	100	200	300	500	700
ZIF-8@ZIF-67/AE-SiC	1	0.971	0.943	0.893	0.806	0.625	0.455	0.357	0.25	0.192

Table S2. Comparison of various adsorbents for Sr^{2+}.

**Materials**	**Equilibrium time (min)**	**pH**	**Adsorption concentration**	**Adsorption capacity (mg/g)**
UNCL[1]	120	10	20 mg/L	7.64
SiSb-0.5[2]	180	3 mol/L HNO_3	100 mg/L	44.30
VSb-0.5[2]	60	3 mol/L HNO_3	100 mg/L	39.93
BiSb-0.1[3]	90	0.1 mol/L HNO_3	100 mg/L	51.69
MIL-100(Fe)-DMA[4]	180	8	500 mg/L	65.82
LTO-MX PAN[5]	480	6.5	10 mg/L	24.05
MnSb[6]	2400	4	10 mg/L	30.2
PVA/GO/MnO_2[7]	4800	7	24.88 mg/L	26.8
mHAP@MnO2[8]	60	7	100 ppm	37.37
**This work**	**30**	**7**	**40 ppm**	**42.7**

Table S3. Comparison of the performance of reported PMS-based catalysts for CIP degradation.

**Catalysts**	**Catalysts dosage**	**PMS dosage**	**CIP concentration**	**Removal efficiency**
$LFPBC_{0.5}$[9]	1 g/L	2 mM	10 mg/L	89.77% (90 min)
5.9Cu-NOC[10]	0.1 g/L	0.5 mM	20 mg/L	100% (30 min)
MNBC[11]	0.8 g/L	0.2 g/L	10 mg/L	92.6% (80 min)
$CuFe_2O_4$/CuO[12]	0.5 g/L	0.3 g/L	5 mg/L	86.67% (120 min)
Co-K-NO-C[13]	0.1 g/L	2 mM	10 mg/L	100% (40 min)
NiCo/Mn/GO[14]	0.15 g/L	0.2 mM	0.02 mM	99% (30 min)
Co_3O_4@PC-HM[15]	0.2 g/L	0.4 g/L	20 mg/L	95% (20 min)
DBC900[16]	2 g/L	2 g/L	20 mg/L	100% (40 min)
**This work**	**0.08 g/L**	**0.4 mM**	**20 mg/L**	**95% (20 min)**

References

1. Tang, X.; Lu, L.; Yang, F.; Zhong, X.; Shi, H.; Zhang, W.; Zhang, L., Recyclable UiO-66-NH_2@Chitosan/Loofah fiber composite for efficient strontium capture. *Sep. Purif. Technol.* **2025,** *379*, 134997.

2. Hu, Y.; Zeng, H.; Yuan, Z.; Wang, W.; Wang, J.; Chen, Q.; Ren, H.; Yan, T., Highly selective and efficient removal of Sr from strong acidic high-level liquid waste using silicon and vanadium-doped antimony oxides. *Sep. Purif. Technol.* **2025,** *353*, 128532.
3. Hu, Y.; Cao, Z.; Yuan, Z.; Wang, W.; Chen, Q.; Ye, G.; Yan, T., Acid-resistant bismuth-doped antimony for effective Sr^{2+} adsorption in nitric acid solutions. *Environ. Res.* **2025,** *276*, 121476.
4. Wang, G.; Zhang, Q.; Qin, L.; Tan, K.; Li, C.; Li, L.; Yang, T.; Liu, X., Construction of MIL-100(Fe)-DMA material for efficient adsorption of Sr and Cs ions from radioactive wastewater. *Sci. Total Environ.* **2024,** *954*, 176296.
5. Lim, Y.; Lee, D. S., Effective radioactive strontium removal using lithium titanate decorated $Ti_3C_2T_x$ MXene/polyacrylonitrile beads. *J. Hazard. Mater.* **2024,** *475*, 134919.
6. Zhang, L.; Wei, J.; Zhao, X.; Li, F.; Jiang, F.; Zhang, M.; Cheng, X., Removal of strontium(II) and cobalt(II) from acidic solution by manganese antimonate. *Chem. Eng. J.* **2016,** *302*, 733-743.
7. Huo, J.; Yu, G.; Wang, J., Efficient removal of Co(II) and Sr(II) from aqueous solution using polyvinyl alcohol/graphene oxide/MnO_2 composite as a novel adsorbent. *J. Hazard. Mater.* **2021,** *411*, 125117.
8. Choi, J.-W.; Lee, H.-K.; Choi, S.-J., Magnetite double-network composite using hydroxyapatite-manganese dioxide for Sr^{2+} removal from aqueous solutions. *J. Environ. Chem. Eng.* **2021,** *9* (4), 105360.
9. Fu, Y.; Yi, Y.; Chen, W.; Wang, Y.; Diao, Z.; Qi, J., Unveiling the critical roles of iron and phosphorus in magnetic biochar derived from lithium-extraction residues of retired $LiFePO_4$ batteries for peroxymonosulfate activation toward ciprofloxacin degradation. *Bioresour. Technol.* **2026,** *448*, 134324.
10. Manpetch, P.; Yang, Y.; Zhou, K.; Xie, R.; Song, W.; Cao, C., Optimal inter-site distance in Cu single-atom catalysts for efficient peroxymonosulfate activation and ciprofloxacin degradation. *Nano Res.* **2026,** *19* (5), 94908537.
11. You, Y.; Zhao, Z.; Song, Y.; Li, J.; Li, J.; Cheng, X., Synthesis of magnetized nitrogen-doped biochar and its high efficiency for elimination of ciprofloxacin hydrochloride by activation of peroxymonosulfate. *Sep. Purif. Technol.* **2021,** *258*, 117977.
12. He, B.; Song, L.; Zhao, Z.; Liu, W.; Zhou, Y.; Shang, J.; Cheng, X., $CuFe_2O_4$/CuO magnetic nano-composite activates PMS to remove ciprofloxacin: Ecotoxicity and DFT calculation. *Chem. Eng. J.* **2022,** *446*, 137183.
13. Liang, J.; Fu, L.; Gao, K.; Duan, X., Accelerating radical generation from peroxymonosulfate by confined variable Co species toward ciprofloxacin mineralization: ROS quantification and mechanisms elucidation. *Appl. Catal. B: Environ.* **2022,** *315*, 121542.
14. Nguyen, T.-B.; Le, V.-R.; Huang, C. P.; Chen, C.-W.; Chen, L.; Dong, C.-D., Construction of ternary $NiCo_2O_4$/MnOOH/GO composite for peroxymonosulfate activation with enhanced catalytic activity toward ciprofloxacin degradation. *Chem. Eng. J.* **2022,** *446*, 137326.

15. Ye, M.; Zhang, H.; Jiang, X.; Jiang, M.; Fan, G., Yeast-biotemplate-assisted fabrication of self-phosphorus doped Co_3O_4@C hollow architecture for ciprofloxacin degradation via peroxymonosulfate activation: Performance, mechanism and toxicity evaluation. *Sep. Purif. Technol.* **2024,** *328*, 125017.
16. Qin, H.; Liu, P.; Hao, J.; Xiao, L.; Wang, Y.; Huang, J.; Chang, J.; Shen, Y.; Xing, B.; Yang, G., Activation of peroxymonosulfate by distillers' grains biochar for the degradation of ciprofloxacin: critical roles of singlet oxygen and electron transfer. *RSC Adv.* **2026,** *16* (23), 20933-20945.
